# How Are Mate Preferences Linked with Actual Mate Selection? Tests of Mate Preference Integration Algorithms Using Computer Simulations and Actual Mating Couples

**DOI:** 10.1371/journal.pone.0156078

**Published:** 2016-06-08

**Authors:** Daniel Conroy-Beam, David M. Buss

**Affiliations:** Department of Psychology, University of Texas at Austin, Austin, Texas, United States of America; Université Toulouse 1 Capitole, FRANCE

## Abstract

Prior mate preference research has focused on the *content* of mate preferences. Yet in real life, people must select mates among potentials who vary along myriad dimensions. How do people incorporate information on many different mate preferences in order to choose which partner to pursue? Here, in Study 1, we compare seven candidate algorithms for integrating multiple mate preferences in a competitive agent-based model of human mate choice evolution. This model shows that a Euclidean algorithm is the most evolvable solution to the problem of selecting fitness-beneficial mates. Next, across three studies of actual couples (Study 2: *n* = 214; Study 3: *n* = 259; Study 4: *n* = 294) we apply the Euclidean algorithm toward predicting mate preference fulfillment overall and preference fulfillment as a function of mate value. Consistent with the hypothesis that mate preferences are integrated according to a Euclidean algorithm, we find that actual mates lie close in multidimensional preference space to the preferences of their partners. Moreover, this Euclidean preference fulfillment is greater for people who are higher in mate value, highlighting theoretically-predictable individual differences in who gets what they want. These new Euclidean tools have important implications for understanding real-world dynamics of mate selection.

## Introduction

Exploring the content of human mate preferences is a well-established research area with both a long history (e.g. [1]) and a cutting edge (e.g., [2]). The downstream consequences of preferences, however, have received comparatively little attention. One critical unanswered question in mate selection is how people incorporate information on many individual mate preferences in order to choose which mates they want to pursue. Humans have many qualitatively distinct preferences which are presumably somehow combined, weighted, or compared in order to make actual mating decisions. Many candidate algorithms could accomplish this computation. Here we use agent-based models of mate choice evolution to test competing hypotheses about which of several algorithms are best able to motivate adaptive mate choice. We then evaluate the ability of the most successful algorithms to explain actual mating data.

Mate preferences are an important part of mate selection across species. Mating is central to reproduction, the primary engine of biological evolution. Mate choice is thus one of the most fitness-impacting decisions a sexually reproducing organism makes [3]. Evolutionary biologists and psychologists expect natural selection to fashion mate preferences in sexually reproducing organisms that act to guide mate choice toward fitness-beneficial mates and away from fitness-costly mates—a premise that has been widely supported in insect, avian, fish, mammalian, and primate species (e.g., [4]).

This premise has also been powerfully supported in evolutionary psychological research. People across cultures show preferences for beneficial qualities like kindness, intelligence, and health as well as sex-differentiated preferences for qualities like age, resource earning potential, and physical attractiveness, consistent with evolutionary hypotheses [5, 6]. These preferences shift contextually, within people, in theoretically principled ways (e.g., mate value, ovulation; [7, 8]), as well as across contexts (e.g., short-term versus long-term; [9, 10]), and across cultures and ecologies (e.g. parasite prevalence; [11]).

The effects of these preferences on human mating outcomes, however, remain unclear. Some studies, primarily using speed-dating methodologies, find that preferences have little if any power to predict real mate choices [12, 13]. Some suggest these weak findings are due to methodological limitations inherent to speed-dating [14, 15]. Another possibility is that the effects of preferences on real mating outcomes are difficult to observe because we lack an understanding of how mate preferences are integrated to make mating decisions [16]. Human mate choice psychology necessarily has mechanisms capable of reducing information on many individual preference dimensions to summary values, such as feelings of attraction, that are useful in making the eventual singular decision of mate selection. Understanding whether and how preferences affect mating outcomes requires understanding the nature of this preference integration process.

The challenge for psychologists is that there exist many viable candidate algorithms by which human mating psychology could integrate mate preferences into overall appraisals. One intuitive algorithm is a linear model similar to linear regression. Mate preferences are hypothesized to evolve because preferred traits are probabilistic indicators of mate value, the fitness benefits a person would offer as a mate [17]. A potentially effective integration psychology could thus estimate mate value as the weighted sum of a potential mate’s traits, with preferences acting as weights proportional to the fitness benefits offered by preferred traits [9, 16]. By multiplying each of a potential mate’s traits by a preference weight and summing across traits, a linear regression algorithm would reduce information about many mate preferences to a single summary attraction value useful in guiding mate search and selection. With a linear regression algorithm, traits that signal more fitness benefits offered could be more strongly preferred, increasing their weighting and contribution to mate value estimates and attraction [9]. If individuals were in turn motivated to pursue potential mates in proportion to this linear combination, a linear regression algorithm could effectively integrate preferences to guide organisms toward adaptive mate choices.

Regression algorithms could also be polynomial in nature. The fitness benefits offered by mates will not necessarily be a linear function of their trait values. It could be, for instance, that either very confident or very humble mates offer large fitness benefits, with intermediate benefits coming from mates who are neither confident nor humble. Additionally, mates incapable of producing shared offspring could offer dramatically fewer fitness benefits relative to mates with even very low fertility [18]. These non-linear effects of traits on fitness are emphasized by the mate preference priority model of preferences [18]. According to this model, the non-linear relationship between trait value and fitness benefits requires preference integration mechanisms that prioritize the acquisition of at least minimum values for some critical traits. Support for this model comes from budget allocation approaches to mate preferences: some traits appear to be treated as “necessities” which are increasingly preferred only until some minimum standard is met; other preferences are “luxuries,” sought in large quantity after necessity preferences are satisfied [19]. To the extent that traits are non-linearly associated with fitness benefits, a simple linear regression model will inadequately integrate mate preferences. The decreasing marginal benefits of necessity traits could require negative quadratic functions: attraction could increase rapidly as a function of trait value before leveling off at an asymptote [20]. Luxuries may require instead a positive quadratic, increasing attraction exponentially as a function of trait value. Non-linearly shaped attraction curves could be produced by polynomial regression algorithms, which weight not only a potential mate’s trait value but also the square of the value, cube of the value, and so on.

Threshold models constitute another set of algorithms that could integrate mate preferences into non-linear attraction curves. With a threshold model, a person would need to pass some pre-set criterion before being considered as a potential mate. By using thresholds, a mate selector could avoid potential mates with highly costly features (e.g. cruel, unhealthy mates) and limit the number of features it needs to consider from each potential mate [16]. For example, a potential mate’s traits might need to fall within some pre-determined range before their traits are entered into weighting functions. Threshold models have been used successfully in predicting mate choice behavior in online dating environments [21]. Miller and Todd [16] proposed a threshold model in which human preference integration psychology employs a sequential aspiration algorithm. With a sequential aspiration algorithm, preferences are integrated not as a linear combination, but as a series of threshold checks. Each preference represents a threshold value to which a potential mate’s traits are compared. If a potential mate surpasses the threshold level on one trait, they pass that aspiration check and are then compared to the next threshold level. Attraction is reserved for people who meet all of a person’s aspirations for a potential mate.

Finally, we propose a novel algorithm that follows directly from the nature of mate selection itself. Each of the algorithms reviewed above ultimately treats mate preferences in isolation. Each trait is compared to its corresponding preference separately, and the results are aggregated across traits. However, potential mates do not present themselves one trait at a time. Rather, potential mates occur as constellations of attributes that must be evaluated and accepted or rejected as a whole [22]. This observation carries a critical logical implication: a potential mate could be an excellent fit to a person’s mate preferences *across* their collection of traits, even if they fail to satisfy any single mate preference in isolation.

A Euclidean integration algorithm can efficiently integrate mate preferences while capturing the inherent multidimensionality of mates and mate choice. A Euclidean algorithm represents potential mates as residing within a multidimensional preference space. Each dimension of this space represents one trait dimension and a potential mate’s position in the space is determined by their trait value on each dimension. Preferences with a Euclidean algorithm represent the ideal value for each trait; consequently, a person’s ideal preferences and their potential mates’ attributes can be located as points within the same multidimensional preference space. Attraction with a Euclidean algorithm can then be computed as the inverse of the straight-line distance between a person’s preference point and each potential mate’s trait point through the multidimensional preference space. This distance is calculated as the sum of the squared deviations between each of a person’s preference values and each of a potential mate’s corresponding trait values. This Euclidean distance can integrate any number of preferences—two, five, 27, and so on—into a single value that represents the overall difference between a person’s constellation of preferences and a potential mate’s constellation of attributes.

Regardless of the merits of each of these algorithms in theory, one fact must be true of the algorithm actually employed by human preference integration psychology: it must have been able to efficiently solve the problem of selecting adaptive mates throughout human evolutionary history. This fact provides researchers one important means of adjudicating among algorithms of mate preference integration.

Agent-based models of mate choice evolution can be used to evaluate an algorithms’ efficacy in selecting adaptive mates. Agent-based models are increasingly employed to study human cooperation (e.g. [23, 24]); though seldom applied, agent-based models can also be useful for evaluating hypotheses concerning human mating psychology. For mate preference integration, algorithms that perform best in simulated evolution are the best candidates for the actual algorithm implemented by human preference integration psychology. Here we construct an agent-based model of human mate choice evolution and compete preference integration algorithms against one another within this model. Across three studies, we then apply the most successful algorithms to three samples of actual mating couples to determine whether they have power in predicting actual mating outcomes.

## Study 1: An Agent-Based Model of Human Mate Choice Evolution

In Study 1, we constructed and analyzed an agent-based model of human mate choice evolution. In this model, agents evolve mate preferences that they use to select other agents as mates. Agents make their mate choice decisions by employing one of seven mate preference integration algorithms: simple linear regression, threshold regression, polynomial regression, aspiration, Euclidean, threshold Euclidean, and random. After mate selection, agents reproduce in proportion to pre-determined fitness values of their traits and their partner’s traits. Agents who make the most adaptive mate choices will better reproduce their traits, preferences, and crucially, preference integration algorithms. By analyzing this model, we can determine which of the candidate algorithms is most efficient at solving the adaptive problem of attracting beneficial mates and therefore which algorithm is the most likely approximation of human preference integration psychology.

### Model

#### Agent generation

700 agents were generated for the parent population. Each agent had a sex, mate preferences, corresponding traits, fitness, and an attraction algorithm. Agents had two possible sexes: male and female. All agents also had 23 traits, initially drawn from random normal distributions with *M* = 4 and *SD* = 1.5 and constrained to values between one and seven. Trait values began normally distributed and uncorrelated, but their distributions and correlational structures were allowed to evolve across generations. The values, correlational relationships, and distributions of traits were thus determined entirely by evolution within the model—aside from trait values being constrained to values between one and seven. Agents were assigned 23 traits in order to be comparable to a mate preference scale under development, not analyzed here. Each agent also had 92 preferences, four per trait, generated initially from random normal distributions with *M* = 0 and *SD* = 1.5. Preferences were allowed to take on any value, positive or negative. What these preference values represented depended on the integration algorithm the agent used. Preferences, like traits, were initially normally distributed and uncorrelated but their ultimate distributions and correlational structures were allowed to evolve across generations. Additionally, like traits, the values, correlational relationships, and distributions of preferences were determined entirely by evolution within the model.

Agents were assigned fitness points based on their traits. For each trait, the model generated a fitness function at the start of each run of the model by assigning to each possible trait value a random whole number between one and seven. This whole number represented the number of fitness points earned by agents with that trait value. No two trait values could be associated with the same number of fitness points. An agent earned from each trait the number of fitness points associated with their trait value; fitness points were then summed across traits. Fitness functions were thus entirely random; results did not change when fitness functions were instead linear or curvilinear (S1 Fig). All traits additionally had the same average impact on fitness; model results did not change when traits contributed differently to fitness (S2 Fig).

For most traits, the same fitness function applied to all agents within the model. However, five traits were sexually dimorphic: fitness functions for these traits were generated separately for males and females and therefore randomly differed between the sexes. The remaining traits were sexually monomorphic and the fitness functions were identical for the sexes. Sexually dimorphic traits were incorporated in order to mimic sex differences found in real mate preference data (e.g. [5, 6, 25]). Five out of 23 traits were chosen to be sexually dimorphic in order to be similar to the proportion of preferences predicted to be sexually dimorphic in mate preference questionnaires (e.g. [5]). However, removing the sexually dimorphic traits did not qualitatively change the model results (S3 Fig).

#### Preference integration algorithm

Each agent was assigned one of seven possible mate preference integration algorithms: simple regression, threshold regression, polynomial regression, Euclidean, threshold Euclidean, aspiration, and random. These algorithms determined how the agent’s preferences were integrated into attraction to other agents. In the parent population, 50 males and 50 females were assigned to each preference integration algorithm. The total population size was 700 agents; manipulating population size did not qualitatively change the results (S4 Fig). Agent preferences evolved unconstrained across generations for all algorithms and thus could take on any value, positive or negative, as well as any distribution or correlational structure.

The simple regression algorithm used the agent’s first preference value for each trait as a slope and the second as an intercept value. This algorithm determined attraction to a given potential mate by multiplying the value of each agent's slope preference by the value of the potential mate's corresponding trait and adding the agent’s intercept preference value. Attraction was the sum of the resulting attraction values across traits. Or $\sum_{1}^{n}p_{1n}*t_{n}+p_{2n}$ where *n* = number of traits, *p* = the agent’s preference value, and *t* the potential mate’s trait value. The third and fourth preference values were not used by this algorithm.

Agents with a threshold regression algorithm first compared their potential mates to a series of threshold checks. Threshold algorithm agents used their third preference value for each trait as an ideal value for that trait and their fourth preference value as an acceptable range around that value. A potential mate passed a threshold check if their trait value fell within the acceptable range around the agent’s corresponding ideal value. For potential mates who passed all threshold checks, attraction was calculated exactly as in the simple regression algorithm. Agents assigned potential mates an attraction value of zero if they failed any threshold check.

The polynomial regression algorithm computed attraction as in the simple regression algorithm, but used their first three preference values for each trait as slopes, allowing construction of a cubic polynomial function. The first slope preference for each trait was multiplied by the potential mate’s trait value, the second by the square of the trait value, and the third by the cube of the trait value. The fourth preference value for each trait served as an intercept. Attraction was the sum of attraction values across traits. Or $\sum_{1}^{n}p_{1n}*t_{n}+p_{2n}*t_{n}^{2}+p_{3n}*t_{n}^{3}+p_{4n}$ where *n* = number of traits, *p* = the agent’s preference value, and *t* the potential mate’s trait value.

The Euclidean algorithm calculated attraction as the inverse of the Euclidean distance between the agent’s first preference value for each trait and the corresponding traits of each potential mate through the multidimensional preference space. Or $1/\sqrt{\sum_{1}^{n}{\left(p_{n}-t_{n}\right)}^{2}}$ where *n* = the number of traits, *p* = the agent’s preference value, and *t* = the agent’s trait value. The three other preferences for each trait were not used by the Euclidean algorithm.

Just as for the threshold regression algorithm, agents with the threshold Euclidean algorithm used their second and third preferences to first compare their potential mates to a threshold check for each trait. Potential mates who failed any threshold check were assigned an attraction value of zero. Potential mates who passed all threshold checks were assigned an attraction value equal to the inverse Euclidean distance between the agent’s first preference for each trait and the potential mate’s corresponding traits.

As in the threshold regression algorithm, agents with the aspiration algorithm had a preferred value and an acceptable range for each trait. Potential mates earned one attraction point for each of their traits that fell within the agent’s acceptable range for that trait. Attraction was calculated as the sum of the attraction points raised to the 15^th^ power. In this way, aspiration agents were only attracted to potential mates who satisfied all or nearly all of their aspirations.

The random algorithm produced a random attraction value for each potential mate drawn from a random uniform distribution ranging from zero to one.

After all agents computed attraction to their potential mates, attraction values were scaled within-algorithm such that the average attraction value for each algorithm was *M* = 50 and the standard deviation was *SD* = 15. This ensured that no algorithm gained an advantage over others due only to the units of its attraction values. Threshold algorithms were scaled such that only above-zero attraction values had a mean of 50 and standard deviation of 15. Attraction values to below threshold mates were maintained as zeros.

#### Life cycle

Within each generation, all agents completed a life cycle wherein they computed their attraction to other agents, selected mates, reproduced, and died.

Agents first went through the “compute attraction” stage of the life cycle. In this stage, each agent computed their attraction to all opposite-sex agents according to their specific algorithms. This stage produced two matrices: one matrix contained the attraction of each female to each male and the other contained the attraction of each male to each female.

After computing attraction, agents entered the mate selection stage. In this stage, the model first computed the mutual attraction of all agents. Pairing based on mutual attraction allowed agents to pair with the mates to whom they were most attracted who were also reciprocally attracted to the agent. This caused mate selection to be attraction-dependent and also captured the fact that human mate choice is mutual and requires attraction from both partners. Mutual attraction was calculated as the element-wise product of the female attraction matrix and the male attraction matrix. The result is the mutual attraction matrix: a matrix of values representing how mutually attracted each possible agent couple would be. The model then completed several steps. (1) The most mutually attracted agents were first determined. These two agents were paired and placed into a matrix of paired agents. (2) The paired agents were removed from the mutual attraction matrix entirely. (3) All agents remaining in the mutual attraction matrix were deducted .25 fitness points. This is a small fitness cost that represents search costs of mating. This cost was applied iteratively, after each pairing event, such that the agents who remained on the mating market longest (i.e. were paired last) paid the largest total search cost. Model results were qualitatively robust to changes in this parameter (S5 Fig). Agents who were left without mates at the completion of the mate selection stage died without reproducing.

Next, paired agents reproduced in proportion to their fitness points. The fitness points of each agent in a pair were summed and divided by 40. The resulting number, rounded down, represented the number of offspring the couple produced. By dividing fitness points by 40, two agents of maximum mate value could produce eight offspring whereas two agents of minimum mate value could produce only one offspring. A reproduction cost of 40 was chosen to produce realistic fertility rates; manipulation of this parameter did not qualitatively change model results (S6 Fig).

The model then initiated the reproduction procedure, which iterated through couples, performing several procedures for each. (1) Each offspring was first assigned a random sex; male and female offspring were equally likely. (2) Offspring received the traits and preferences of their parents, depending on their sex. For the five sexually dimorphic traits, offspring inherited the trait values and corresponding preferences of their same-sex parent. For the sexually monomorphic traits, offspring had an equal chance of inheriting each trait value and preference from either parent. (3) Each offspring inherited their preference integration algorithm from their parents. Offspring had an equal chance of inheriting the algorithm of either parent. (4) Once generated, offspring mutated. Random normal noise, centered on *M* = 0 with *SD* = .5 was added to each trait and preference value; manipulating the amount of noise added during mutation did not qualitatively change the results (S7 Fig). Algorithms and sex could not mutate. Traits were constrained to values between one and seven after mutation. After mutation, the fitness values of offspring were calculated exactly as for their parents.

After all couples reproduced, the offspring matrices were saved over the parent matrices and the couple matrices were emptied. Additionally, if the offspring population size was larger than *n* = 700, a random sample of offspring was erased such that the population size remained at *n* = 700. Capping the population size served both to maintain computability of the simulation and introduce realistic forces of genetic drift.

At this stage, a full life cycle was completed and the offspring generation began their life cycle. The model ran for 200 generations of evolution. Model script is available in the supporting materials (S1 Script).

### Results

**Fig 1:**
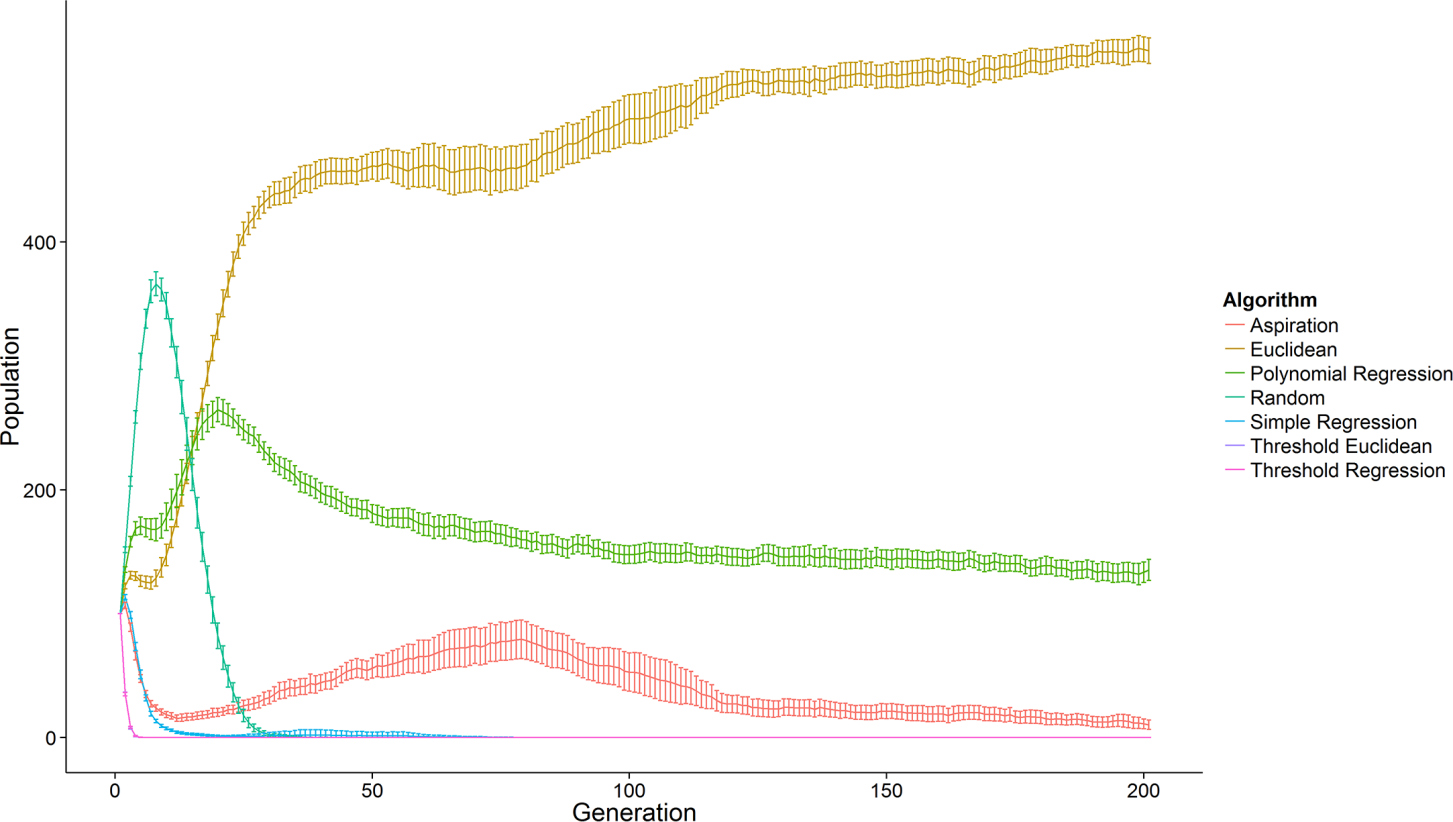
Population size of each algorithm across generations based on 50 total model runs. Population size is the average population of each algorithm at each generation across model runs. Error bars represent 95% confidence intervals.

We ran our agent-based model 50 times in total. Within each run of the model, we saved the population size of each algorithm at each generation. We aggregated the generation-level population sizes across model runs and saved the average population size as well as 95% confidence intervals for each algorithm at each generation. Fig 1 plots the population sizes for algorithms across generations. Table 1 presents the performance of each algorithm across model runs including the proportion of runs in which each algorithm was (1) dominant (had the largest population size), (2) fixed (eliminated all competitor algorithms), and (3) extinct by the final generation.

**Table 1:** Performance of each mate preference integration algorithm across model runs.

	Proportion of model runs in which each population was…		
	Dominant	Fixed	Extinct
Euclidean	50 (100%)	0 (0%)	0 (0%)
Polynomial Regression	0 (0%)	0 (0%)	0 (0%)
Aspiration	0 (0%)	0 (0%)	20 (40%)
Random	0 (0%)	0 (0%)	50 (100%)
Regression	0 (0%)	0 (0%)	50 (100%)
Threshold Euclidean	0 (0%)	0 (0%)	50 (100%)
Threshold Regression	0 (0%)	0 (0%)	50 (100%)

The simple regression, threshold regression, and threshold Euclidean models went extinct almost immediately in all model runs. The random algorithm showed surprisingly good performance in the early generations. In these early generations, the randomness of this algorithm did not appear to be a substantial cost because the principled algorithms had not yet evolved effective, functional mate preferences. During this period the random algorithm did not make substantially poorer mate choices but did pay fewer search costs, perhaps because their idiosyncratic attraction values meant they experienced less competition for mates on average than did other algorithms. The random algorithms were eventually and quickly outcompeted by the principled algorithms once the latter evolved functional mate preferences.

The longest surviving algorithms were the polynomial regression, aspiration, and Euclidean algorithms. The aspiration algorithm frequently persisted in the final generation, but went extinct in 40% of model runs. The polynomial algorithm never went extinct, but was also never the dominant algorithm.

The most successful preference integration algorithm across model runs was by far the Euclidean algorithm. Across model runs, the population size of this algorithm increased rapidly and occupied the population size made available by the extinction of the regression algorithms. This algorithm never fixed, but was also dominant in 100% of model runs and never went extinct. Its average population size in the final generation, *M* = 554.36, was 4.10 times greater than the average population size of the next most successful algorithm, the polynomial algorithm (*M* = 135.26).

### Discussion

We compared seven candidate preference integration algorithms to one another in a competitive agent-based model of human mate choice evolution. This model captured several key adaptive problems posed by real human mate choice including competition among mating rivals and the challenge of securing mutual attraction. But crucially, agents had an array of mates to choose from, each of whom offered fitness benefits that were opaque to the agent. Solving this adaptive problem required evolving ideal mate preferences and integrating them in such a way that agents were able to reliably choose the most fitness beneficial partners. Algorithms that more effectively solved this problem were favored by selection and became more numerous within our simulated population.

The most successful model in our algorithm was by far the Euclidean algorithm. Two key features of this algorithm contribute to its success. First, the Euclidean algorithm is the only algorithm capable of representing potential mates as the multidimensional individuals that they are. This algorithm is able to motivate attraction to mates who represent good fits to mate preferences overall rather than to mates who merely fit individual preferences. Second, the algorithm is extremely efficient in its parameter usage. The Euclidean algorithm is capable of computing a multidimensional attraction variable using only one parameter per trait. This makes the Euclidean algorithm able to quickly evolve functional preferences and maintain these preferences against mutation over evolutionary time. The next simplest algorithm, the simple regression algorithm, required two parameters per trait: twice as many parameters that must first be evolved and then maintained across generations.

The success of the Euclidean algorithm within our model suggests that Euclidean preference integration would have been successful in solving the adaptive problem of attracting beneficial mates throughout human evolution. Nonetheless, it is important to stress that a model is only good as its assumptions. Although we manipulated many of our assumptions, we could not manipulate all of them. For example, the Euclidean algorithm was able outcompete the alternative algorithms we tested, but there may exist other algorithms that could perform better. Additionally, threshold models performed poorly within our model but may be more competitive in models where fitness is distributed less continuously across trait ranges. Agents within our model were able to assess all potential mates on all traits and paid no penalties for using more information; models that incorporate more restricted search may favor different algorithms, such as those that use thresholds. Agents also made mating decisions individually, without influence of kin, friends, or rivals. Our agents were purely monogamous, even though humans apply a mix of mating strategies including short-term mating [19] and extra-pair mating. Finally, it is possible that people apply combinations of algorithms: for instance, using thresholds for some traits and continuous integration for others.

Future research could continue to apply our model to explore more algorithms under further varying conditions. However, it would be impossible to explore all algorithms under all possible sets of assumptions. What is clear based on our model is that, among many algorithms for integrating mate preferences, the Euclidean algorithm succeeds robustly across a wide range of circumstances. This success suggests that human preference integration psychology is particularly likely to employ an algorithm similar to the Euclidean algorithm. Thus, the next critical test of this algorithm concerns its ability to explain features of real human mate choice. In Studies 2–4, we sought to provide evidence that human preference integration psychology employs a Euclidean algorithm by evaluating whether a Euclidean approach to mate preferences has power in predicting real mating outcomes.

## Study 2: Applying Euclidean Distances in Newlywed Couples

Study 1 suggested that a Euclidean method is a particularly evolvable candidate algorithm for integrating individual mate preferences into overall appraisals of potential mates. This suggests that actual human preference integration psychology is likely to integrate mate preferences using a Euclidean algorithm. Agent-based models again provide a useful means to test this hypothesis. If human preference integration psychology employs a Euclidean method, trends produced by agent-based models of Euclidean mate choice should approximate corresponding trends found in samples of real human couples. Further, agent-based models employing other integration algorithms should not approximate human trends as well as do Euclidean models.

One particularly important trend is preference fulfillment: the extent to which actual mates fulfill ideal mate preferences. A Euclidean algorithm operates by motivating selection of mates whose traits minimize the Euclidean distance from one’s ideal preferences. If mate preferences are integrated according to a Euclidean algorithm, actually chosen mates should fall close to ideal preferences within the multidimensional preference space. Here we test this prediction by examining the relationship between mate preferences and partner traits in agent based models of Euclidean-based mate choice, polynomial and aspirational mate choice, and in a sample of actual newlywed couples. If human preference integration psychology does actually employ a Euclidean method, newlywed couples should show multidimensional preference fulfillment and this preference fulfillment should be similar to that observed in agent-based models of Euclidean preference integration but not polynomial or aspirational integration.

### Method

#### Participants

We analyzed data from a sample of 214 partners composing 107 heterosexual newlywed couples. Participants were recruited by mail from public marriage records in a large county in the Midwestern United States. All couples had been married for less than one year at the time of participation. The average age for male participants was 26.68 (SD = 3.71); females were 25.54 years old on average (SD = 4.05). All participants provided written consent to participate in this study. This study was approved by the University of Michigan Institutional Review Board.

#### Measures

For each participant, we analyzed ratings on 40 7-point bipolar adjective pairs (e.g. “submissive” to “dominant”) representing the five factors of personality. Adjective pairs were chosen as the highest-loading items from Goldberg [26]. This instrument included many traits that people are known to value in potential mates including “intelligent”, “agreeable”, and “generous.” Ratings for each trait come from self-report, partner-report, and the report of two independent interviewers. All ratings were averaged together to create composite scores. For personality preferences in a mate, participants rated their desired partner personality on the same set of bipolar adjective scales.

#### Model

The models in Study 2 proceeded as in Study 1 with two changes. First, we began with an initial population of only 100 agents and a population cap of 200. This lowered population size increased computability of the simulation while still giving a large enough population size for data analysis. Second, agents in each model used only one preference integration algorithm: either Euclidean, polynomial, or aspiration.

In order to calculate Euclidean distances between ideal preferences and partner traits, we saved from the Euclidean model the traits, preferences, and partners of all agents in the final generation of the model. Within the polynomial and aspirational models, we inferred the agents’ ideal preference values based on the preference functions they evolved. For polynomial agents, we presented each agent with all possible trait values for each trait and determined which trait value caused the agent to return the largest attraction value. These values were saved as their ideal preferences for each of the 23 traits. For aspirational agents, their ideal preference for each trait was determined as the center of their acceptable range for that trait.

#### Data analysis

**Fig 2:**
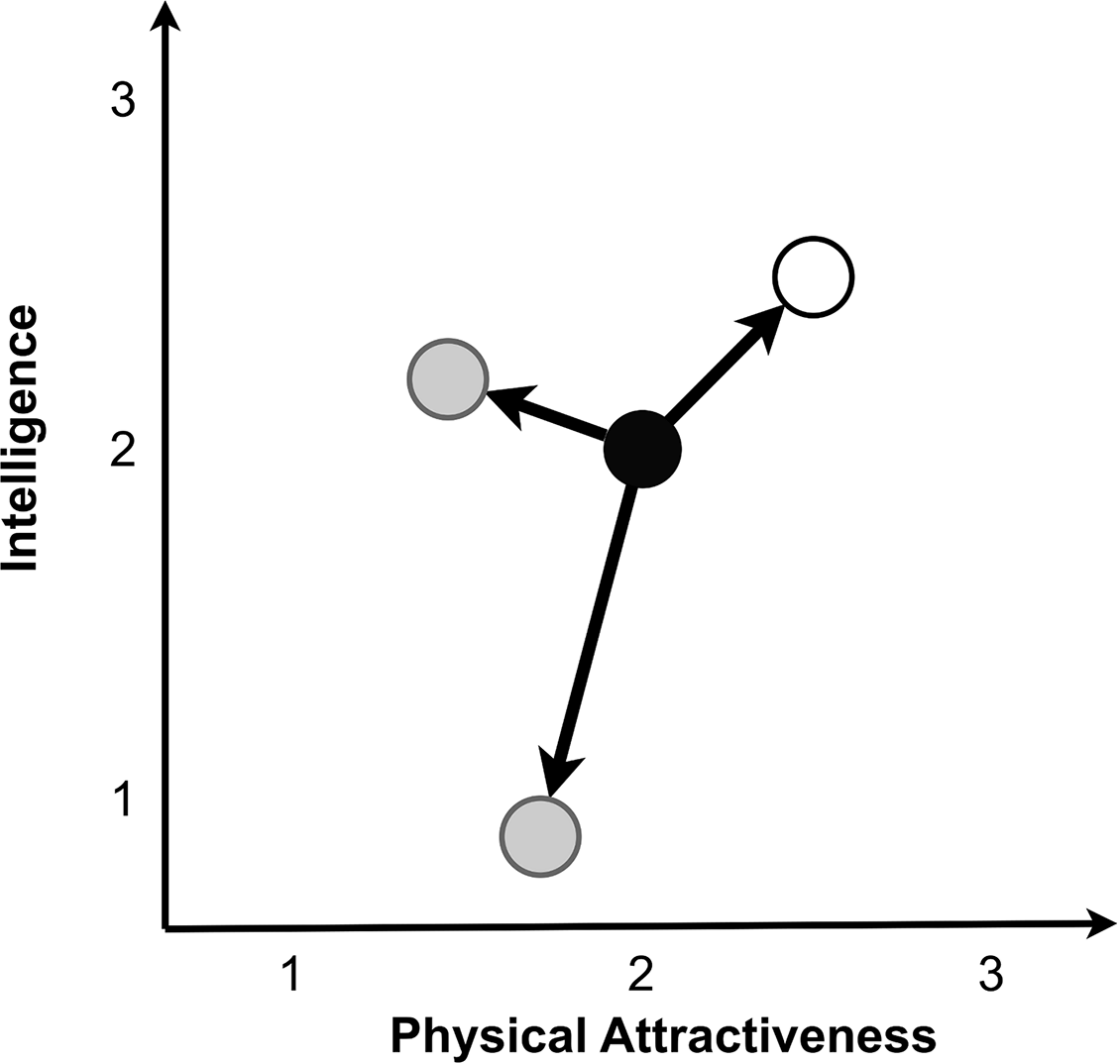
The Euclidean distance method for quantifying preference fulfillment in a small hypothetical population. A person’s preferences (black) and be plotted in a preference space alongside their partner’s qualities (white) and the qualities of their alternative potential partners (grey).

Within the models and the newlywed sample, we first calculated the Euclidean distance between each participant’s or agent’s ideal preferences and their partner’s corresponding traits. Fig 2 provides a graphical representation of the Euclidean distance analysis for just two mate preferences and qualities. A person’s mate preferences can be plotted in an *n*-dimensional space where each axis represents a trait and location on the axis represents trait value. A partner’s actual qualities can also be plotted in this preference space. The Euclidean distance between ideal preferences and partner traits is the linear distance between a person’s preference point and their partner’s trait point.

Raw Euclidean distances from the models and newlywed sample are not directly comparable because they are based on different numbers of dimensions: 23 dimensions within the models and 40 dimensions within the newlywed sample. To allow comparison between the newlywed sample and the models, we scaled Euclidean distances from the newlywed sample to their equivalent values within a 23-dimensional space.

Euclidean distances between preferences and partner traits provides an *absolute* index of preference fulfillment: lower distances indicate a participant or agent’s mate provides a good absolute fit to their preferences. But even a partner who is far from preferences absolutely could be a good mate if they are closer to preferences than are alternative mates. We therefore calculated for each agent and participant an index of *relative* preference fulfillment as the proportion of alternative mates within the sample or model who were further from that agent or participant’s preferences than their actually selected mate. A larger value of this percentage means that this agent or participant’s mate is closer to their preferences than most mates and indicates their mate is a good *relative* fit to their preferences.

We ran each agent-based model 50 times in total and calculated absolute and relative preference fulfillment within each model run. Results for each are reported as the average degree of preference fulfillment across model runs with 95% confidence intervals based on the standard deviation in preference fulfillment across model runs. For the newlywed sample, we bootstrapped confidence intervals on preference fulfillment by resampling with replacement from the newlywed couples 10,000 times. Results are reported as the average preference fulfillment across bootstrap samples with 95% confidence intervals representing the values that cut off the most extreme 2.5% of samples in both directions.

### Results

#### Absolute preference fulfillment

Agents within the Euclidean model ultimately selected mates that were on average a Euclidean distance of *M* = 11.25 units away from their ideal preferences, 95% CI [11.11, 11.38]. Euclidean agents’ mates were closer to their preferences than mates selected by polynomial agents (*M* = 13.35, 95% CI [13.14, 13.56]) and mates selected by aspirational agents (*M* = 19.97, 95% CI [19.73, 20.22]). Participants within the newlywed sample were mated to partners who were very close to their preferences on average: *M* = 6.58, 95% CI [6.55, 6.61]. Absolute preference fulfillment within the newlywed sample was thus greater than preference fulfillment within any of the agent-based models, but was most similar to preference fulfillment observed within the Euclidean model.

#### Relative preference fulfillment

Agents within the Euclidean agent-based model selected mates who were on average closer to their preferences than 87.29% of alternative mates, 95% CI [86.87, 87.71]. Agents in the polynomial agent-based model selected mates closer to their preferences than just 53.03% of alternative mates on average, 95% CI [52.34, 53.71]. Aspirational agents selected mates closer to their preferences than 57.38% of alternatives, 95% CI [56.71, 58.04]. Relative preference fulfillment was surprisingly weak within the newlywed sample: participants were mated to partners who were closer to their preferences than just 60.96% of alternatives, 95% CI [60.37, 61.54]. Despite strong absolute preference fulfillment, in terms of relative fulfillment participants in the newlywed sample were most similar overall to aspirational agents. All data are available in the supporting materials (S1 Data).

### Discussion

The newlywed couples showed only partial correspondence with the agent-based model of Euclidean mate selection. Newlywed couples show strong absolute preference fulfillment—much stronger than observed within the agent-based models, but most similar overall to that observed in the aspirational model. However, newlywed couples showed relatively weak relative preference fulfillment most similar overall to aspirational agents.

Some amount of the strength of relative preference fulfillment in the Euclidean agent-based model is unsurprising. Agents in the agent-based model are able to perfectly appraise their mates’ traits, compute attraction without error, and pursue mates in direct proportion to attraction. Real mate selection is unlikely to proceed as ideally, even if it is Euclidean in nature, due to a number of constraints, such as unreliability of assessment about a potential mate’s traits due to lack of relevant information, probabilistic rather than certain cue validity, deceptive information, or low observability about potential mates’ actual trait values (e.g., [14, 15, 27]). The most important difference, however, is that in the agent-based model, all mate preferences were important to mate selection. The preferences and qualities assayed in the newlywed sample pertain exclusively to personality traits and are missing variables common to mate preference research such as age, physical attractiveness, or resources. Although people appear to acquire mates with desirable personality traits absolutely, these traits may simply not be important enough for people to strive for strong relative preference fulfillment. Thus, we conducted Study 3 in order to replicate the findings of Study 2 but also attempt to address its key limitation by examining preferences more common to mate preference research.

## Study 3: Applying a Euclidean Approach in an Internet Sample of Long-term Couples

In Study 3, we applied the Euclidean distance assay of preference fulfillment to agent-based models and an internet sample of people in long-term relationships. The online nature of this study allows for potentially broader sampling than available in newlywed sample of Study 2. Additionally, we focus analysis on a set of preference dimensions more central to theoretically-driven mate preference research than the personality dimensions analyzed in Study 2.

### Methods

#### Participants

Participants were 259 people (140 female) recruited from Amazon’s Mechanical Turk. All participants reported being in ongoing, heterosexual, committed long-term relationships. Of these, 148 were married, 88 reported dating exclusively, 4 reported being engaged, 1 reported “living together,” and 22 reported “dating casually”. The average relationship length was *M* = 90.65 months (*SD* = 105.85). The average age for female participants was 35.22 (*SD* = 11.38); males were 34.34 years old on average (*SD* = 10.83). Participants consented to this study by submission of an online consent form. This consent procedure and this study were approved by the University of Texas at Austin Institutional Review Board.

#### Measures

Participants completed the mate preference questionnaire from Buss [5] with some modifications. Participants completed the questionnaire with the original 18 dimensions as well as nine added dimensions: dominant, confident, intelligent, masculine, feminine, muscular, kind, mutually attracted, and age difference. These traits were included because they are commonly explored in the broader mate preference literature. Participants rated the importance of these traits in their ideal long-term partner on 7-point Likert scale ranging from “Irrelevant (Extremely unimportant)” to “Indispensable (Extremely important)”. Participants separately rated the extent to which these traits described their actual long-term partner using 7-point Likert scales ranging from “Strongly disagree” to “Strongly agree.” Participants additionally reported the actual age difference between themselves and their partners in years. Some participants (*n* = 19) misunderstood the question asking for ideal age difference as asking for ideal age of partner. This resulted in, for instance, 25-year-old participants stating they ideally preferred partners 20 years younger than themselves. We eliminated from analyses participants that clearly misunderstood the instructions in this way.

#### Model

The agent-based models proceeded exactly as in Study 2.

#### Data analysis

Data analysis proceeded exactly as for Study 2.

### Results

#### Absolute preference fulfillment

As in Study 2, agents within the Euclidean agent-based model selected mates that were on average *M* = 11.19 units away from their mate preferences, 95% CI [11.04, 11.34]. This was closer than mates selected by polynomial agents, *M* = 13.17, 95% CI [12.96, 13.39], and mates selected by aspirational agents, *M* = 20.08, 95% CI [19.83, 20.34]. Participants in the long-term couples sample were mated to mates closer to their preferences on average than observed in any agent based model, *M* = 7.37, 95% CI [7.30, 7.43], but most similar overall to absolute preference fulfillment observed within the Euclidean model.

#### Relative preference fulfillment

Euclidean agents selected mates closer to their preferences than *M* = 87.62% of alternatives, 95% CI [87.19, 88.05]. This relative preference fulfillment was stronger than that observed in the polynomial model, *M* = 52.82%, 95% CI [52.33, 53.31], and in the aspirational model, *M* = 56.86%, 95% CI [56.33, 57.39]. In contrast to Study 2, participants within the long-term couple sample showed strong relative preference fulfillment much more comparable to relative preference fulfillment within the Euclidean model. Participants were mated to partners closer to their preferences than 83.30% of alternatives, 95% CI [82.87, 83.73].

All data is available in the supporting materials (S2 Data).

### Discussion

Study 3 replicates the results of Study 2 in finding that people’s actual mates fall a short Euclidean distance from their ideal preferences in absolute terms—closer than observed in any agent-based model, but still most similar to that observed within the Euclidean model. The results of Study 3 additionally suggest substantially stronger relative preference fulfillment than the results of Study 2. Actual partners lie closer to preferences than to the overwhelming majority of alternative partners. This preference fulfillment is importantly much more comparable to that found in the agent-based model of Euclidean mate choice.

Part of these stronger preference fulfillment effects in Study 3 relative to Study 2 may be due to the inclusion of more important mate preference dimensions, such as physical attractiveness. Rater variance could be an additional cause. Ratings in Study 3 were based solely on participant self-report; preferences therefore share rater variance with qualities of chosen partners but not of alternative partners. It is possible, for instance, that participants favorably perceive the traits of their own partners as better matching their preferences more than do third party raters, causing Study 3 to overestimate preference fulfillment. However, re-analysis of Study 2 did not show systematically larger effects from self-reports than from composites including partner and interviewer reports. In fact, absolute and relative preference fulfillment were both *weaker* overall in the newlywed sample when based on self-report alone than when based on composite ratings. Thus, it appears more likely that Study 2 *underestimated* preference fulfillment in being based on less important mate preference dimensions.

## Study 4: Euclidean Preference Fulfillment and Mate Value in Long-term Couples

Study 4 had two goals. First, the effects of Study 3 using mate preference variables were surprisingly large in comparison to the effects found for personality variables in Study 2. Study 4 was therefore conducted to determine whether the relatively large effects of Study 3 were replicable.

Second, Studies 2 and 3 showed that Euclidean distances have power in predicting mating outcomes in that simulated agents and real long-term mates show a modest to large degree of preference fulfillment in both relative and absolute terms: mates lie absolutely close to ideal preferences through the preference space and are closer on average than alternative partners. Study 4 attempted to extend this predictive power in showing that Euclidean distances can also predict individual differences in preference fulfillment.

Studies 2 and 3 do suggest that actual mates do fulfill ideal mate preferences, but theoretically this preference fulfillment should be constrained by multiple factors. One important factor is a person’s own desirability as a mate: people who are themselves more desirable can attract more potential mates and therefore should have more power in selecting mates who fulfill their preferences. People more desirable on the mating market should therefore on average tend to be mated to partners who better match their ideal mate preferences.

Euclidean distances can be used to test this hypothesis in two ways. First, Euclidean distances can be used to quantify the degree of absolute and relative preference fulfillment for a given person as in Studies 2 and 3. Second, Euclidean distances can also be used to quantify a person’s overall desirability as a mate—their “mate value.” If mate preferences are truly integrated using a Euclidean algorithm, the most desirable people will be those whose traits tend to fall close in multidimensional preference space to the preferences of the opposite sex. A person’s mate value can thus be calculated as the Euclidean distance between their traits and the average mate preferences of the opposite sex.

The hypothesis that ideal mate preferences are integrated according to a Euclidean algorithm therefore makes two key predictions: (1) peoples’ actual mates should fall closer to their preferences in multidimensional preference space both absolutely and relatively as in Studies 2 and 3, and (2) preference fulfillment should be correlated with the Euclidean distance between a person’s traits and the preferences of the opposite sex, such that people who better embody the preferences of the opposite sex in Euclidean terms tend to be mated to people who better fulfill their own ideal preferences. Here we test these predictions in agent-based models and a third sample of actual mated couples.

### Method

#### Participants

Participants were 294 members of long-term couples (129 female) recruited using Amazon’s Mechanical Turk. Female participants were 32.88 years old on average (*SD* = 10.03); males were 35.04 years old on average (*SD* = 10.71). All participants were in long-term heterosexual relationships: 17 reported dating casually, 72 dating exclusively, 31 engaged, 171 married, and 3 reporting “other” relationship statuses: “separated”, “living together”, and “long-term partnership”. Participants had been in their relationships for *M* = 68.99 months on average (*SD* = 86.30). A small set of participants (*n* = 7) misunderstood the question asking for ideal age difference as asking for ideal age of partner. Participants who misunderstood this question were excluded from analyses. Participants consented to this study by submission of an online consent form. This consent procedure and this study were approved by the University of Texas at Austin Institutional Review Board.

#### Measures and Model

Participants completed the same measures as in Study 3. In addition to rating their ideal and actual partners, participants also rated themselves on the mate preference dimensions, excluding relationship specific items such as “mutual love”, on 7-point Likert scales ranging from “Strongly Disagree” to “Strongly Agree”. The agent-based model proceeded exactly as in Study 2.

#### Data Analysis

We measured preference fulfillment exactly as in Studies 2 and 3. Mate value was calculated as the inverse of the Euclidean distance between each agent’s and each participant’s own traits and the corresponding average ideal preferences of the opposite sex. A higher value on this index therefore indicated the agent or participant better embodied the ideal preferences of the opposite sex overall. All data are available in the supporting materials (S3 Data).

### Results

Participants in Study 4 were again mated to partners very close to their ideal preferences on average, *M* = 7.08, 95% CI [7.01, 7.15]. This absolute preference fulfillment was more similar to that observed within the Euclidean models (*M* = 11.03, 95% CI [10.92, 11.5]) than to absolute preference fulfillment in the polynomial models (*M* = 13.13, 95% CI [12.89, 13.36]) or the aspirational models (*M* = 20.06, 95% CI [19.78, 20.34]). Relative preference fulfillment from participants in Study 4 (*M* = 81.04%, 95% CI [80.59, 81.50]) was similarly strong to relative preference fulfillment in the Euclidean models (*M* = 87.53%, 95% CI [87.02, 88.05]). Relative preference fulfillment was weaker in the polynomial models (*M* = 52.78%, 95% CI [52.68, 53.67]) and aspirational models (*M* = 57.34%, 95% CI [56.84, 57.84]).

**Fig 3:**
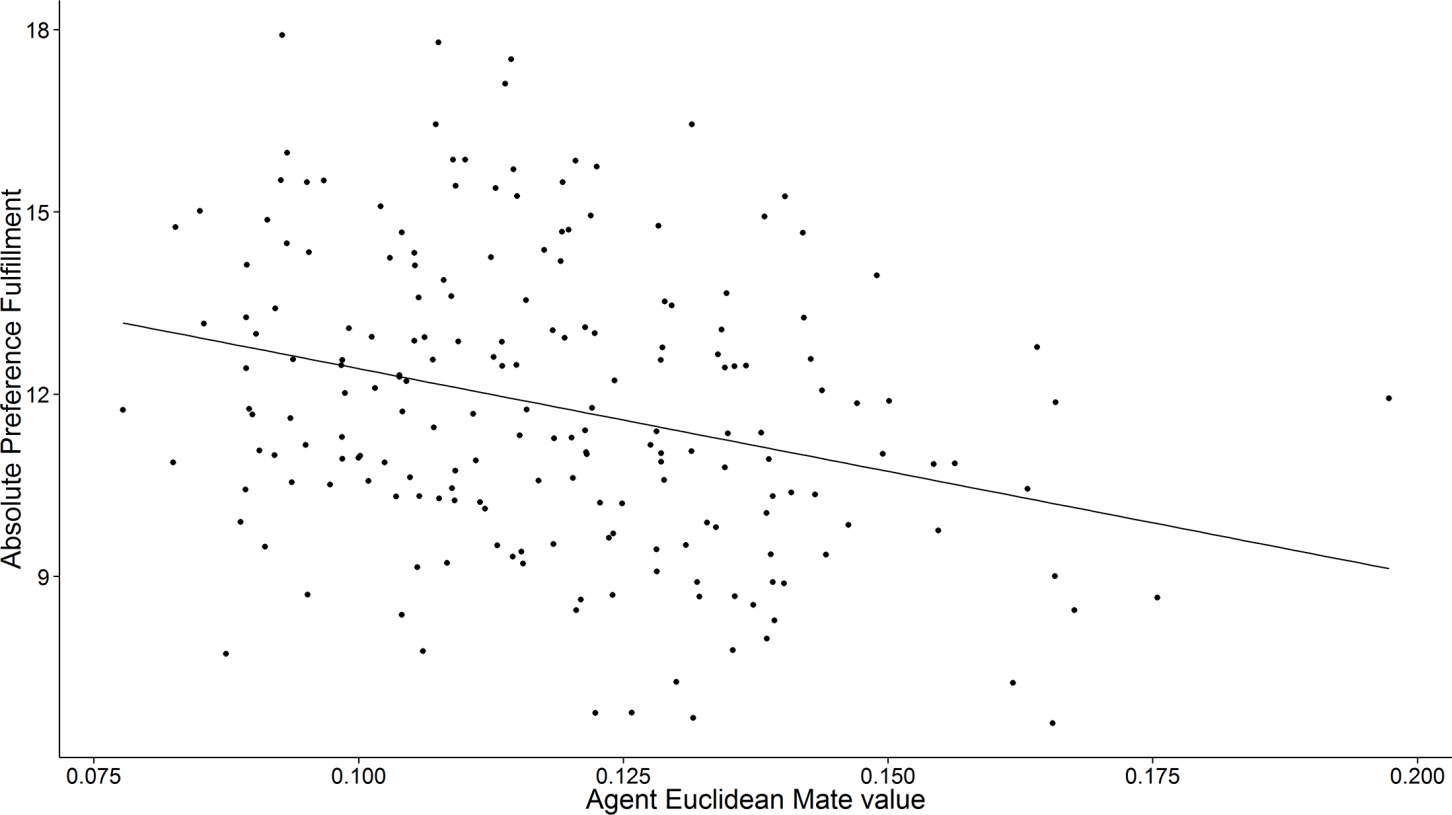
Absolute preference fulfillment as a function of agent mate value within one run of Study 4’s Euclidean agent-based model. Consistent with predictions, agents who are themselves more desirable as mates are on average mated to partners who better fulfill their ideal mate preferences.

Fig 3 plots an example of the relationship between agent mate value and preference fulfillment from one run of the Euclidean agent-based model. Consistent with predictions, agent mate value was negatively correlated with preference fulfillment across runs of the Euclidean model, *r* = -.17, 95% CI [-.19, -.14]. Agents who were themselves more desirable as mates tended to attract and be mated to partners who were closer to their ideal mate preferences through the multidimensional preference space. This negative correlation between mate value and preference fulfillment is also uniquely predicted by Euclidean preference integration: this relationship did not emerge in models in which agents integrate their preferences according to a polynomial algorithm (*r* = .01, 95% CI [-.02, .04]) or in which agents use an aspirational algorithm (*r* = .13, 95% CI [.09, .17]).

**Fig 4:**
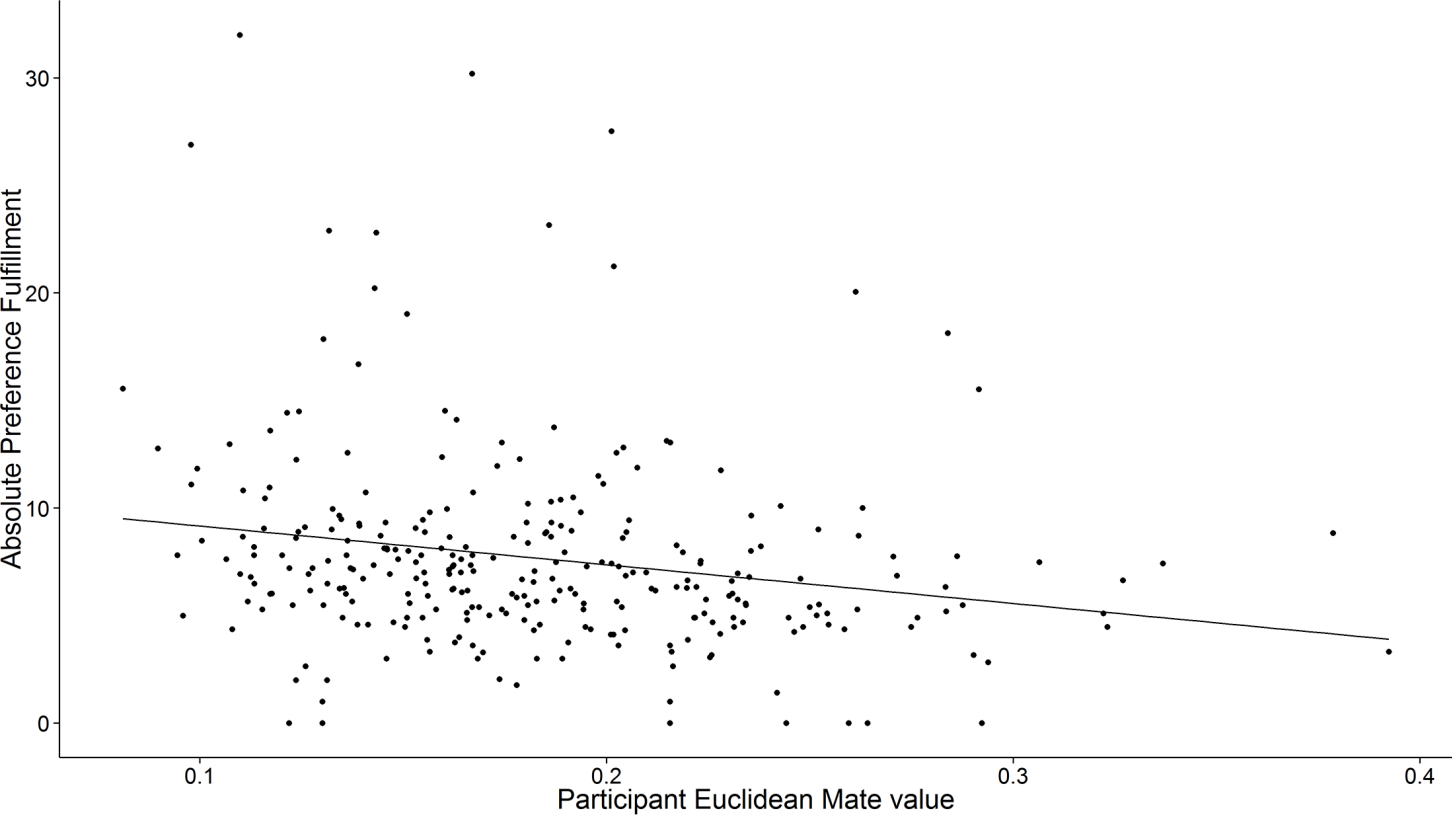
Preference fulfillment as a function of participant mate value. Participants in Study 4 who better embodied the preferences of the opposite sex were mated to partners who better fulfilled their ideal mate preferences.

Fig 4 shows that the negative relationship between preference fulfillment and mate value emerged within the long-term couple sample. Participants who were higher in mate value according to a Euclidean calculation were mated to partners who were closer to their mate preferences through the multidimensional preference space, *r* = -.21, 95% CI [-.23, -.19].

### Discussion

Study 4 offered two key findings. First, in terms of preference fulfillment, Study 4 robustly replicated the effects of Study 3. Using 27 dimensions more traditional to mate choice literature, Study 4 produced similarly large effects as Study 3—in fact, actual mates in Study 3 were closer fits to preferences than approximately the same percentage of alternative mates: 81.04% on average in Study 4 relative to 83.30% on average in Study 3. Average absolute preference fulfillment in Study 4 (7.08) was also very similar to that observed in Study 3 (7.37). These preference fulfillment effects were once again very comparable to preference fulfillment observed in the agent-based model of Euclidean mate selection. These large, replicable effects whereby actual mates provide good fits to ideal mate preferences in multidimensional space serve as important evidence that human mate preference psychology employs something like a Euclidean algorithm to integrate mate preferences in making mating decisions.

Second, Study 4 provided support for the hypothesis that people higher in mate value themselves, as calculated using Euclidean distances, will have partners who better fulfill their ideal mate preferences. The ability of higher mate value people to acquire partners closer to their mate preferences is theoretically consistent, but to our knowledge has not previously been demonstrated. This novel finding has two key implications. First, the correlation between mate value and preference fulfillment supplies further evidence that human mate preferences are integrated in mate choice using a Euclidean algorithm. This relationship is uniquely predicted by Euclidean preference integration in that it does not emerge from models wherein agents integrate their preferences according to polynomial or aspirational algorithms. Crucially, this effect emerges both within models of Euclidean preference integration and our sample of long-term couples.

Second, this finding further speaks against the potential limitation of Studies 3 and 4 being based on self-report. Any degree of preference fulfillment within our samples of human couples could be explained by participants deluding themselves about the proximity of their mate preferences to their partners’ traits. However, this participant delusion process would not predict that high mate value would delude themselves more than low mate value people. In fact, the opposite might have been predicted: because low mate value people are likely to acquire partners further from their mate preferences, one could argue that they might be more motivated to convince themselves that their preferences have been fulfilled. The correlation between mate value and preference fulfillment observed in both the Euclidean agent-based model and the long-term couple sample suggest that the correspondence between ideal mate preferences and partner traits is unlikely to emerge because of participant self-deception. Study 4 suggests that the most likely scenario is that people select their mates by integrating their preferences according to a Euclidean algorithm.

## General Discussion

Four studies provide evidence that a Euclidean algorithm is a strong candidate for the algorithm by which human mate preferences are integrated in mate choice. Among a group of seven candidate algorithms, the Euclidean algorithm proved to be the most evolvable strategy by far for selecting mates in our agent-based model of human mate choice evolution. However, theoretical models are only as good as their assumptions. It is crucial, then, that our agent-based models converged across three samples with empirical data on real mating couples to support the Euclidean algorithm as the best description of human mate preference integration. In three samples of human couples, people’s mate preferences fall close in multidimensional space to their actual partner’s qualities: close absolutely and closer than to the majority of alternative possible partners. Finally, only the Euclidean algorithm furnished a key novel finding: people who are themselves higher in mate value are able to leverage their desirability into selecting mates who better fulfill their ideal preferences. Together, the findings of four studies provide important new evidence that human mate preferences are integrated by a psychology employing a Euclidean algorithm.

The success of the Euclidean algorithm appears to come from several sources. First, the Euclidean algorithm is able to easily integrate many mate preferences into singular decision variables capable of guiding mate choice. Second, this efficient integration comes from using relatively few parameters: just one for each trait. Finally, and perhaps most importantly, the Euclidean algorithm matches the multidimensional nature of mate choice: a mate’s mate value is derived from consideration of all of their qualities simultaneously, rather than mere summation of their traits independently. This means that the impact of any one trait on a person’s overall mate value depends on the value of all of their other traits. That is, possessing one highly undesirable trait can have a relatively large impact on the mate value of an otherwise desirable person but only a small impact on a person who is undesirable on several other dimensions.

A greater understanding of the algorithm by which mate preferences are integrated will be essential for advancing research on the downstream consequences of mate preferences [16]. Preference research has made much progress in discovering the content of human desire, especially those elements of desire that can be consciously articulated (e.g., [5, 6]). Our novel Euclidean method allows researchers to apply information about the content of participants’ preferences toward computing summary values of preference fulfillment, mate value, and even mate value discrepancies. These summary values provide the first tractable method for widespread study of the connections between mate preferences and actual mating outcomes. Future research could use our Euclidean preference fulfillment index in experimental contexts or alongside other individual differences to further understand the determinants of who succeeds and who fails in attracting their ideal partners. Beyond mate selection, Euclidean distance variables could be used to assess the within-relationship consequences of succeeding or failing to acquire mates who fulfill one’s mate preferences for outcomes such as relationship satisfaction, relationship longevity, and probability of relationship dissolution. These novel findings would furnish a greater understanding of human mating psychology, but also provide further evidence bearing on the hypothesis that human mate preferences are integrated according to a Euclidean algorithm.

It is important to stress that our findings show that a Euclidean preference integration algorithm is consistent with human mate choice data but does not provide direct evidence that human preference integration psychology actually employs a literal Euclidean algorithm. First, mate preference fulfillment is a key finding consistent with Euclidean preference integration, but predicting attraction would be an even more direct test of the Euclidean algorithm. If human mate preferences are integrated according to a Euclidean algorithm, Euclidean distances between a person’s ideal preferences and the traits of potential mates should predict that persons’ attraction to those potential mates. Future research employing Euclidean distances to predict attraction prior to relationships would provide important evidence bearing on human preference integration.

Additionally, just as a snail needs no mathematical ability to construct the perfect logarithmic spiral of its shell [28], human mate choice psychology could in principle be designed to act *as though* it employed a Euclidean algorithm without necessarily calculating Euclidean distances. Euclidean preference fulfillment alone is not sufficient evidence to rule out this alternative hypothesis. Future research must continue to apply Euclidean distances to predict and explain mating phenomena. The greater diversity of mating phenomena a Euclidean algorithm is able to predict, the more confident researchers can be that human preference psychology actually employs a Euclidean algorithm to integrate mate preferences.

Furthermore, although models employing Euclidean preference integration were able to produce mate selection findings similar to findings revealed in actual studies of couples, this fit was clearly imperfect. Relative preference fulfillment within the Euclidean agent-based models was stronger than the same preference fulfillment within samples of human couples. Absolute preference fulfillment within the human couple samples was stronger in all samples than in any of the agent-based models. These results suggest a Euclidean algorithm is likely a good approximation of the algorithm applied by human preference integration psychology, but more realistic algorithms may nonetheless be able to produce more realistic data. For instance, the Euclidean algorithm assumes that all mate preferences are equally important—surely an unrealistic assumption. Algorithms that are able to incorporate the differential importance of preferences may be able to produce mating data more similar to real human data. Future research can continue to compare agent-based models and data from real couples to refine our understanding of the design features of human preference integration psychology.

In addition to evidence bearing on mate preference integration, the results of the current study also establish that mate preference fulfillment is substantial yet clearly imperfect. The reasons for lack of fit may themselves prove just as interesting a topic for future research as do the determinants of good fit. Mate preferences do not operate in a vacuum. Imperfect preference fulfillment is plausibly attributable to many forces and constraints present within real mating markets. Selecting a mate requires evaluating an array of potential mates on an array of features and identifying potential mates who best match one’s preferences. However, mating rivals are doing the same. Each person must compete to attract and be chosen by desirable mates. Most people necessarily will not be able to succeed for at least three reasons: (1) because ideal partners may not exist in available mating pools, (2) because their ideal partners choose to mate with competitors, and (3) because a person’s own mate value constrains their ability to translate desires into actual successful reciprocal mate selections. The relatively modest effect of mate value on preference fulfillment is a testament to the importance of these many dynamics: multiple constraints act to limit a person’s preference fulfillment, meaning each is likely to have only modest effects. Determining the relative contributions of these dynamics, inherent to real mating markets, to both the match and mismatch between preferences and obtained partners is essential for understanding the behavioral effects of human mate preferences.

Preference fulfillment in our samples was sometimes substantial, yet interpreting this preference fulfillment is made difficult by the fact that preferences and partner traits were collected at the same time. Correspondence between variables could emerge, as in our model, because preferences guide mate selection. But they could also emerge if people merely adjust their mate preferences to match the traits of their selected partners. Some prior research suggests this explanation is unlikely; for instance, mate preferences are generally consistent over time [29]. The findings of our study, too, speak against this explanation as *post-hoc* preference updating could not explain the tendency of higher mate value people to be mated to partners closer to their mate preferences. Nonetheless, longitudinal data on mate preferences, particularly before and after mate choices, would be ideal for determining the causal source of mate preference fulfillment. Agent-based modeling would also be a good tool for evaluating this possibility in future research.

A final implication of this research centers on the key construct of mate value. Euclidean distances provide the first objective, quantitative method for estimating mate values. Mate value as a concept has been the target of much theorizing (see [17]) but empirical research has been hampered by the lack of an effective operationalization of this key construct. With Euclidean distances, researchers can calculate the match between a person and the preferences of individual potential mates, the preferences of all available potential mates, or the preferences of specific groups of potential mates (e.g., those pursuing a short-term vs. long-term mating strategy). These Euclidean distances estimate a person’s mate value overall, to particular people, or in particular circumstances—each of which may importantly moderate different aspects of human mating psychology. Study 4 established the first evidence that such Euclidean calculations can predict real mating outcomes in theoretically sensible ways: people who are high in Euclidean mate value, and thus well-embody the preferences of the opposite-sex, are able to leverage that mate value into attracting mates who better fulfilled their preferences. This is novel finding that reveals that desirability on the mating market is an important determinant of real mate choice outcomes. But even further, this critical finding both provides unique evidence suggesting mate preferences are integrated according to a Euclidean algorithm and establishes the utility of using a Euclidean approach to measure mate value. Continuing to pair the established predictive power of evolutionary functional hypotheses and the new methodological opportunities offered by the Euclidean distance measure could go far toward closing the gap between understanding the content of human mate preferences and their multiple downstream mating effects, including relationship satisfaction, within-relationship conflict, infidelity, longevity, and dissolution.

## Supporting Information

S1 Data Study 2 data. Newlywed couple and agent-based model data analyzed in Study 2.
(ZIP)

S2 Data Study 3 data. Long-term couple and agent-based model data analyzed in Study 3.
(ZIP)

S3 Data Study 4 data. Long-term couple and agent-based model data analyzed in Study 4.
(ZIP)

S1 Fig Simulation results as a function of fitness function manipulation. Agent-based model results employing either linear fitness functions (A) or curvilinear functions (B). For linear models, the model randomly determined whether the function for each trait would be positive or negative. Fitness points associated with each trait value increased by one from one to seven for positive functions and decreased by one from seven to one for negative functions. For curvilinear functions, the model generated a vector of fitness point values that increased from one to seven by two and then decreased back from seven to one. This vector was shifted rightward by a random number of positions for each trait, resulting in a random curvilinear fitness function for each trait.
(TIFF)

S2 Fig Simulation results in models where traits contribute differently to fitness. Agent-based model results based on a model in which traits do not have equivalent contributions to fitness. Fitness functions were generated by scrambling vectors of fitness points both within and between traits such that some traits could earn more fitness points overall than other traits.
(TIFF)

S3 Fig Simulation results in models where all traits are sexually monomorphic. Agent-based model results wherein all traits are monomorphic.
(TIFF)

S4 Fig Simulation results as a function of manipulating total population size. Agent-based model results employing population sizes of 350 agents (A) or 1,050 agents (B).
(TIFF)

S5 Fig Simulation results as a function of manipulating search costs. Agent-based model results employing search costs of .125 (A) and .375 (B).
(TIFF)

S6 Fig Simulation results as a function of manipulating reproduction costs. Agent-based model results employing reproduction costs of 20 (A) and 60 (B).
(TIFF)

S7 Fig Simulation results as a function of manipulating mutation rate. Agent-based model results employing mutation rates of .25 (A) and .75 (B).
(TIFF)

S1 Script Simulation script. R script for running and analyzing the agent-based models.
(DOCX)
