# Supplementary figures and images for: How Are Mate Preferences Linked with Actual Mate Selection? Tests of Mate Preference Integration Algorithms Using Computer Simulations and Actual Mating Couples
Source: PLoS One. 2016 Jun 8;11(6):e0156078. doi: 10.1371/journal.pone.0156078 (PMC4898694; doi:10.1371/journal.pone.0156078)

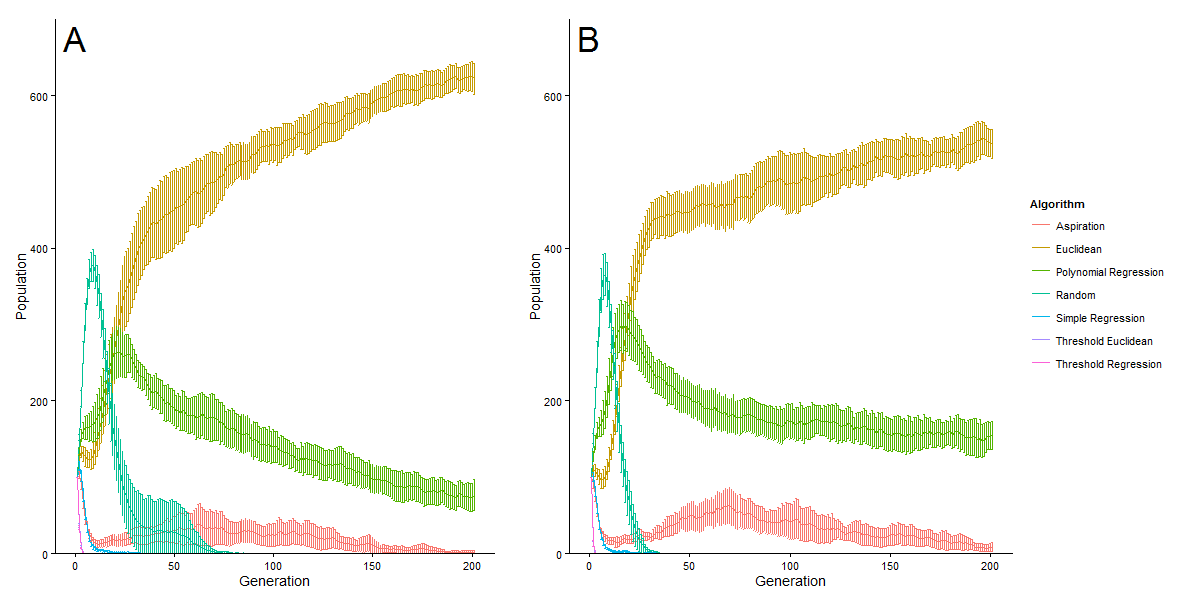

Supplement: S1 Fig — Agent-based model results employing either linear fitness functions (A) or curvilinear functions (B). For linear models, the model randomly determined whether the function for each trait would be positive or negative. Fitness points associated with each trait value increased by one from one to seven for positive functions and decreased by one from seven to one for negative functions. For curvilinear functions, the model generated a vector of fitness point values that increased from one to seven by two and then decreased back from seven to one. This vector was shifted rightward by a random number of positions for each trait, resulting in a random curvilinear fitness function for each trait. (TIFF) [file pone.0156078.s004.tiff]

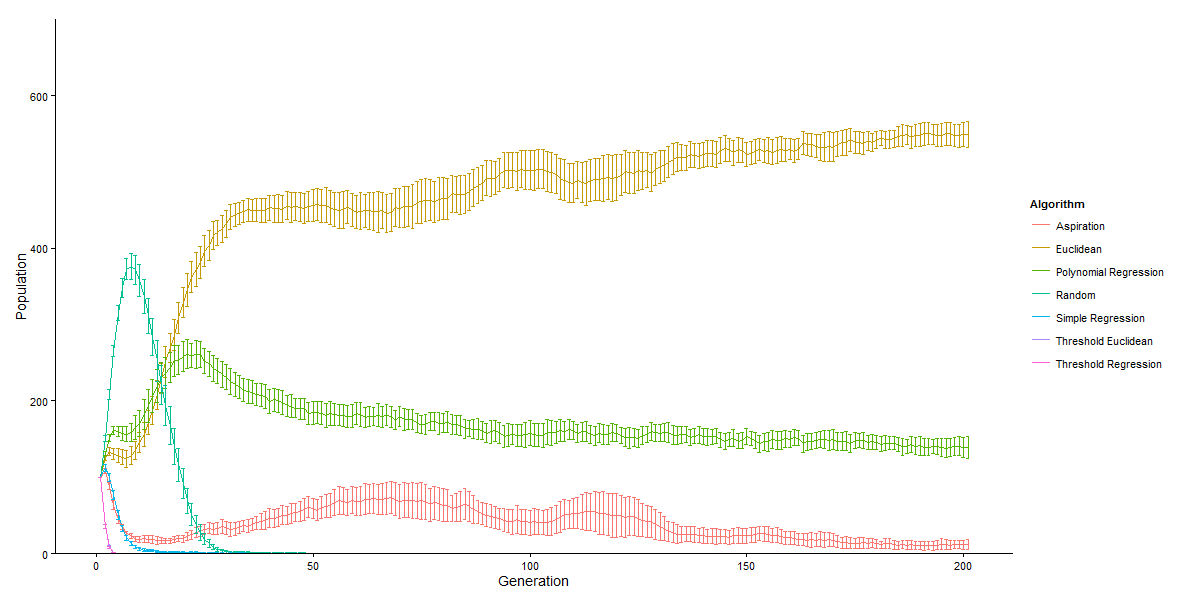

Supplement: S2 Fig — Agent-based model results based on a model in which traits do not have equivalent contributions to fitness. Fitness functions were generated by scrambling vectors of fitness points both within and between traits such that some traits could earn more fitness points overall than other traits. (TIFF) [file pone.0156078.s005.tiff]

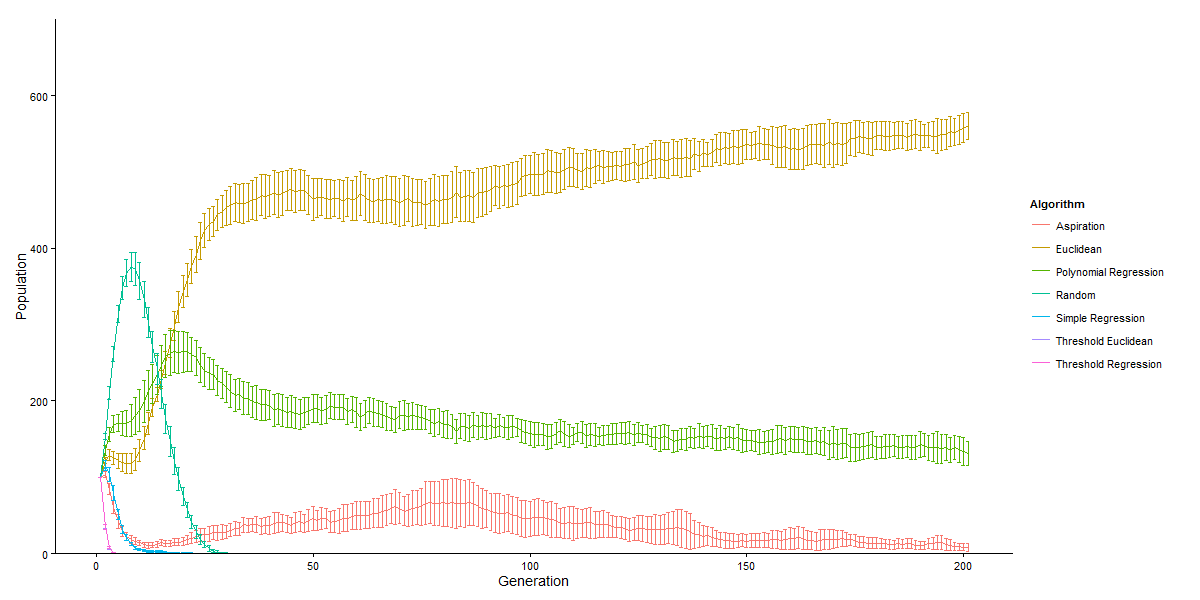

Supplement: S3 Fig — Agent-based model results wherein all traits are monomorphic. (TIFF) [file pone.0156078.s006.tiff]

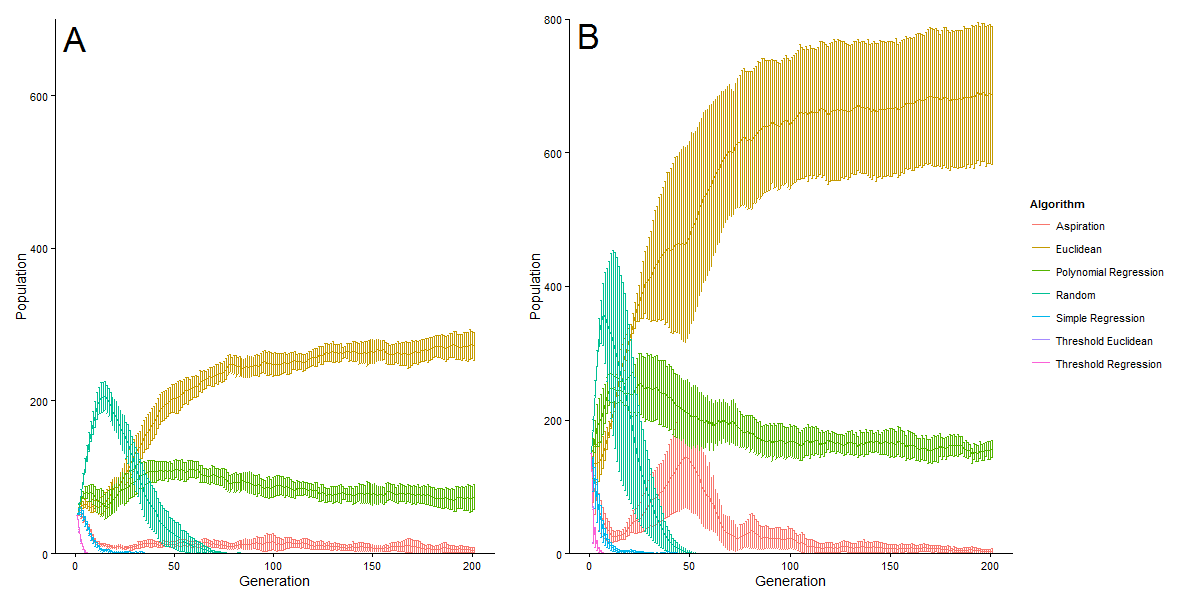

Supplement: S4 Fig — Agent-based model results employing population sizes of 350 agents (A) or 1,050 agents (B). (TIFF) [file pone.0156078.s007.tiff]

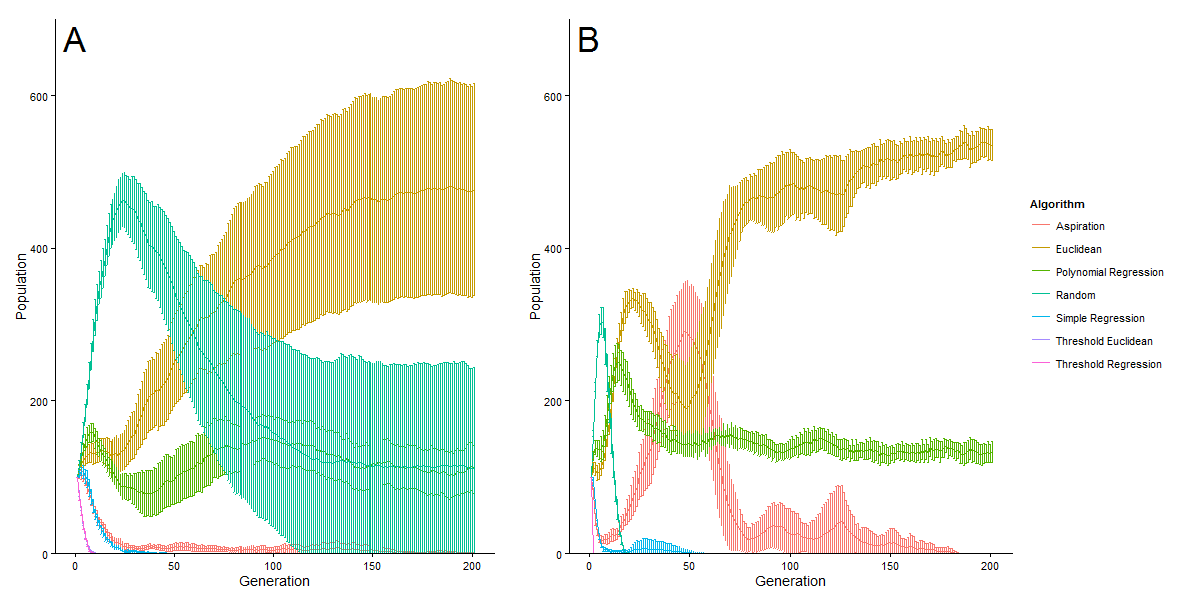

Supplement: S5 Fig — Agent-based model results employing search costs of .125 (A) and .375 (B). (TIFF) [file pone.0156078.s008.tiff]

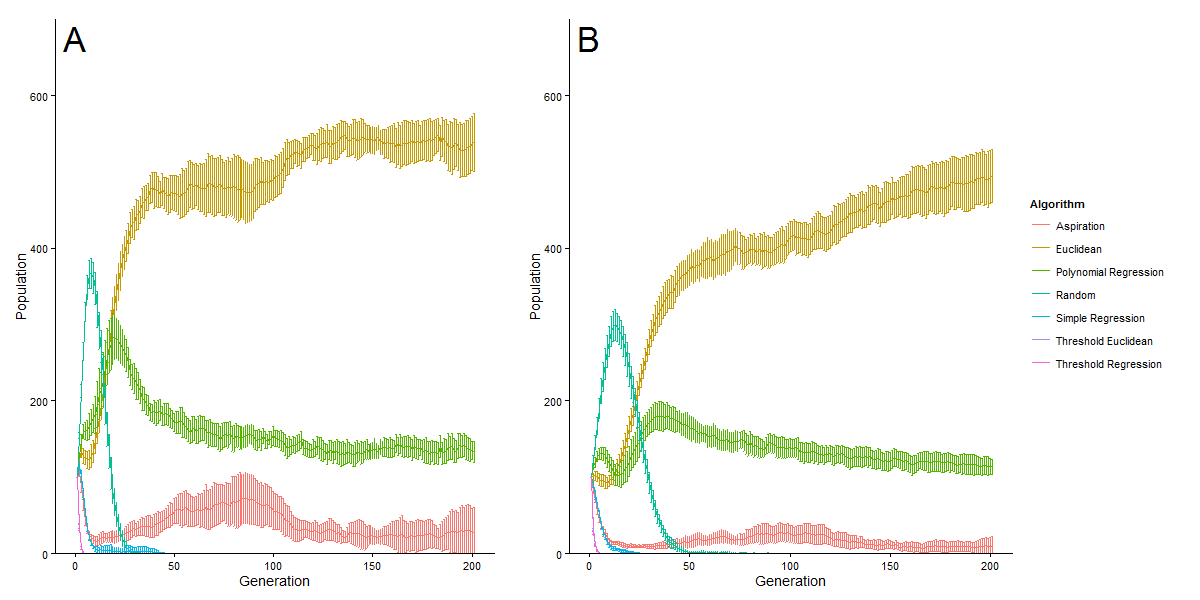

Supplement: S6 Fig — Agent-based model results employing reproduction costs of 20 (A) and 60 (B). (TIFF) [file pone.0156078.s009.tiff]

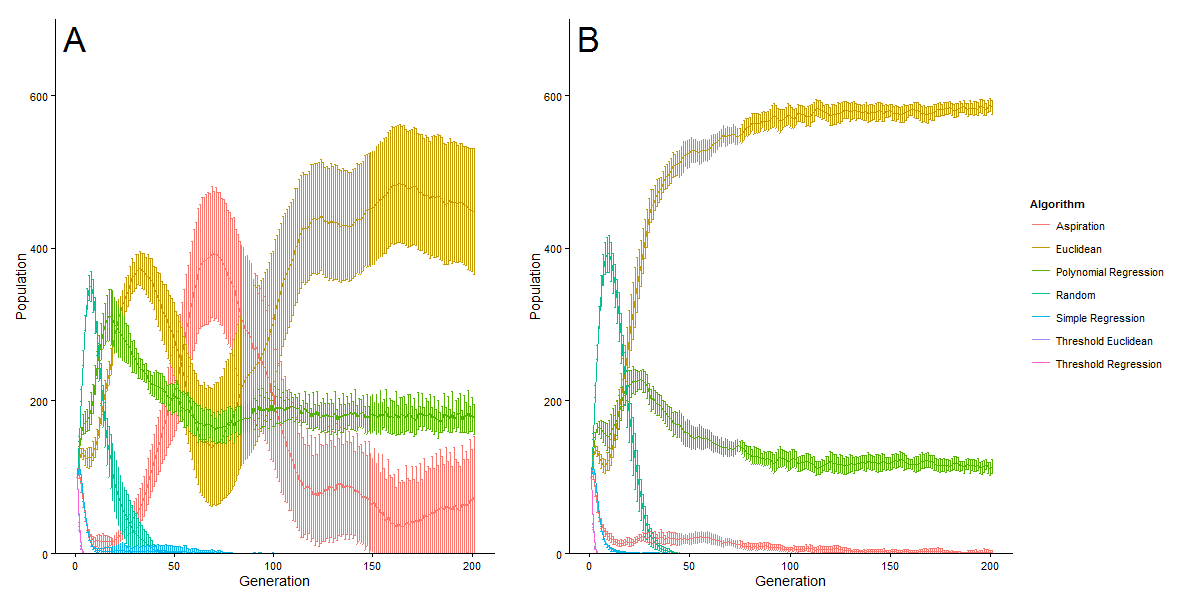

Supplement: S7 Fig — Agent-based model results employing mutation rates of .25 (A) and .75 (B). (TIFF) [file pone.0156078.s010.tiff]
