# Supplementary material for: How Are Mate Preferences Linked with Actual Mate Selection? Tests of Mate Preference Integration Algorithms Using Computer Simulations and Actual Mating Couples
Source: PLoS One. 2016 Jun 8;11(6):e0156078. doi: 10.1371/journal.pone.0156078 (PMC4898694; doi:10.1371/journal.pone.0156078)
Supplement: S1 Script — R script for running and analyzing the agent-based models. (DOCX) [file pone.0156078.s011.docx]

# Evolution of Mate Choice Simulation

#

# ###Purpose###

# An agent based model of mate choice incorporating reproduction and sexual selection. This model will allow me to manipulate and observe mate preference driven mate choice in a population of simulated agents. From this I will be able to test hypotheses about how mate selection affects sexual selection and how sexual selection affects mate choice. The hope is to create a fairly modular, basic simulation that can be modified to explore several questions in mate selection. My immediate goal for this simulation is to observe which of several potential mate preference integration algorithms is most evolvable.

#

# ###Agent description###

# 700 agents will be generated. Each agent will have mate preferences and corresponding traits, a unique PIN, fitness points, and an attraction algorithm.

#

# Traits and preferences

# Each agent will have 23 traits. Traits will be constrained between 1 and 7. Traits will be generated from random normal distributions.

# Agents will also have preferences. Each agent will have 92 preferences, 4 per trait. For most agents, not all preferences will matter. Preferences will be unconstrained numerics drawn from random normal distributions.

# All agents will have a unique PIN value.

#

# Fitness

# Each agent will have fitness points. Fitness points will be based on the agent's traits. 5 traits will be sexually dimorphic and will be assigned different fitness vectors for each sex. The remaining traits will be sexually monomorphic and will have identical fitness vectors between the sexes.

# The fitness vector will be produced by first creating vectors of 1-7 for each trait and then randomly scrambling each vector. Each trait value will thereby be associated with a random fitness value.

# For each agent, the fitness points earned will be determined by comparing the value of each of their traits to the fitness vector for those traits. For each trait, the agent will earn the number of fitness points associated with their trait value in that trait's fitness vector. The agent's total fitness points will be the sum of the fitness points earned from each trait.

# These fitness points will be spent to have offspring. The fitness points of each member of a couple will be summed and converted into offspring. 40 fitness points will equal one offspring. That way, two agents of maximum mate value could have 8 offspring in total.

#

# Attraction algorithm

# Each agent will have one of seven attraction algorithms. 100 agents will be assigned to each attraction algorithm; 50 males and 50 females.

# Simple regression. This algorithm will determine attraction to a given potential mate by multiplying the value of each agent's preference by the value of the potential mate's corresponding trait and adding an intercept value. Attraction will be the sum of the resulting attraction values across traits.

# Threshold regression. Agents with this algorithm will compare their potential mates to a series of threshold checks. Threshold algorithm agents will have both an ideal value for each trait and an acceptable range around that value. A potential mate will pass a threshold check if their trait value falls within the acceptable range around the agent's corresponding ideal value. For potential mates who pass all threshold checks, agents will calculate attraction exactly as in the simple regression algorithm. Potential mates who fail any threshold check will be assigned an attraction value of zero.

# Polynomial regression. Attraction according to this algorithm will be computed exactly as for the non-threshold regression. The agent will multiply each of their preferences by the traits of each potential mate and sum across traits. However, the agent will have 4 preferences per trait that will be added and multiplied by potential mates traits using a cubic regression formula: p1*t + p2*t2 + p3*t3 + p4. These agents can therefore have curved attraction functions for each trait.

# Euclidean. Each agent will calculate the Euclidean distance between their preferences and the traits of their potential mates. Attraction will be the inverse of this value.

# Threshold Euclidean. Just as for the threshold regression algorithm, agents with threshold Euclidean algorithm will first compare their potential mates to a threshold check for each trait. Potential mates who fail any threshold check will be assigned an attraction value of zero. Potential mates who pass all threshold checks will be assigned attraction values equal to the inverse Euclidean distance between the agent's preferences and the potential mate's traits.

# Aspiration. Agents using this algorithm will have two preferences per trait. These preferences will establish an acceptable range of trait values. For each potential mate, the agent will determine whether each of the potential mate's traits fall within that agent's acceptable range. For each trait within the agent's acceptable range, a potential mate will be assigned 1 attraction point. Attraction will be calculated as the sum of these attraction points raised to the 15th power. In this way, aspiration agents will only be attracted to potential mates who satisfy all or nearly of their aspirations.

# Random. This algorithm will assign random attraction values to potential mates that will be independent of both the agent's preferences and the potential mate's traits.

#

# After all agents compute attraction, attraction values will be scaled within algorithm. Each algorithm's attraction values will be scaled such that the mean attraction value is 50 and the standard deviation is 15. This prevents algorithms being favored simply because their attraction values are on a different scale of measurement. Attraction values of 0 will be preserved and excluded from scaling for agents with threshold algorithms.

#

# ###Life cycle###

# 1. Compute attraction

# 2. Select mates

# 3. Reproduce

# 4. Die

#

# Computing attraction

# Each agent will compute their attraction to all other agents according to their specific algorithm.

#

# Mate selection

# The simulation will next compute the mutual attraction matrix for all agents. This is the product of attraction values for all possible couples. The selection procedure will next follow several steps.

# (1) The most mutually attracted couple will first be determined. These two agents will be paired and placed into a separate matrix of paired agents.

# (2) Each agent that was not paired in that iteration will first lose .25 fitness points. This is a small cost that represents search costs of mating. This cost will be applied to all unpaired agents regardless of their traits, algorithm, or attraction values. The cost will be applied on each iteration, setting up a selection pressure for mates who are able to choose and be chosen early.

# (3) The selection procedure will remove the paired male and female from the mutual attraction matrix.

# This process will iterate until all possible pairs are made. If there are agents who cannot pair with a mate (e.g. odd number of agents sex ratios), these agents will not be placed in the paired agent matrix.

#

# Reproduction

# Paired couples will reproduce in proportion to their fitness points. The fitness points of each parent will be summed together and then divided by 40. The resulting number, rounded down to the nearest integer, will represent the number of offspring that couple produces.

# Next, two offspring matrices will be generated for storing the offspring of the couples: one for male offspring, the other for female offspring. The reproduction procedure will then iterate through couples, performing several procedures for each.

# For each couple, the reproduction procedure will produce each of their offspring one at a time.

# (1) Each offspring will first be given a random sex. Females will be coded as 0, males as 1.

# (2) Offspring will receive traits and preferences from their parents, depending on their sex. For sexually dimorphic traits, offspring will inherit the trait values and associated preferences for that trait from their same-sex parent. For seuxally monomorphic traits, offspring will have a 50% chance of inheriting each preference and trait value from either parent.

# (3) Each offspring will have a 50% chance of inheriting the algorithm of either parent.

# (4) Once offspring are produced, they will mutate. The model will add random normal noise to each of the offspring agent's trait and preference values. This noise will be centered on 0 with SD = .5. Sex and algorithm will not mutate.

# (5) Next, the fitness points of the offspring will be determined using the same procedure as was used for parents.

# (6) Finally, the created offspring will be stored in the correct matrix for their sex.

#

# Death

# After all agent couples reproduce, the offspring matrices will be saved over the parent matrices. The couple matrix will also be emptied. This effectively kills all parents. Additionally, if the offspring population size is larger than 700, a random subset of offspring will be killed such that the population size remains at 700.

# Capping the population size has a couple of nice features: (1) it keeps the simulation computable (population size would increase exponentially otherwise, exponentially decreasing computability) and (2) it introduces realistic forces of genetic drift.

# After death, surviving offspring will be assigned unique PINs.

# At this stage, the full life cycle is complete. The next generation will start at the beginning of the life cycle. Evolution will continue for 200 generations.

######Functions######

#Fitness calculation#

fitnesspointscalc<-function(agenttraits,fitnessvector){

#agenttraits is the traits of an individual agent

#fitnessvector is the fitness vector for that agent's sex

#Determines and sums the fitness points associated with the agent's traits

return(sum(diag(fitnessvector[round(agenttraits,0),])))

}

#Agent generation#

agentgenerate<-function(popsize,sex,algorithm){

#Prevents problems if you try to generate a population with a population size of 0

if(popsize>0){

#Traits are drawn from random normal distribution. Traits are named appropriately and constrained to be between 1-7

traits<-apply(matrix(,popsize,23),2,function(x) rnorm(popsize,4,1.5))

colnames(traits)<-paste(paste('trait',1:ncol(traits),sep=""))

traits[traits>7]<-7

traits[traits<1]<-1

#Preferences are generated unconstrained from a random normal distribution

preferences<-apply(matrix(,popsize,92),2,function(x) rnorm(popsize,0,1.5))

colnames(preferences)<-paste('preference',1:ncol(preferences),sep="")

#Fitness points are calculated for generated agents

if(sex==1){

fitpoints<-apply(traits,1,function(x) fitnesspointscalc(x,malefitvec))

}

if(sex==0){

fitpoints<-apply(traits,1,function(x) fitnesspointscalc(x,femalefitvec))

}

sex<-sex

algorithm<-algorithm

return(as.data.frame(cbind(traits,preferences,fitpoints,sex,algorithm)))

}

}

#Attraction calculation#

attraction<-function(agentprefs,agentalgorithm,targettraits){

#agentprefs is the ideal preferences of an individual agent

#agentalgorithm is the algorithm of that individual agent

#targettraits is the traits of all opposite-sex agents

#Algorithms:

#1 = regression

#2 = aspiration

#3 = polynomial regression

#4 = threshold regression

#5 = euclidean

#6 = threshold euclidean

#7 = random

#Attraction for regression algorithm

if(agentalgorithm == 1){

#Multiplies the agent's preferences by each mate's traits, adds an intercept, and sums across traits

return(apply(targettraits,1,function(x) sum(x*agentprefs[seq(1,92,4)]+agentprefs[seq(2,92,4)])))

}

if(agentalgorithm == 2){

#For each trait, the agent checks whether the potential mate's trait value is within the acceptable range. A potential mate gets an attractiveness point for each aspiration check they pass.

aspiration<-sapply(1:23,simplify=T,function(x) abs(as.numeric(agentprefs[((x*4)-3)])-targettraits[,x]) <= abs(as.numeric(agentprefs[((x*4)-2)])))

#Overall attraction is the number of levels passed raised to the 15th power. This makes it so that the agent is only attracted to a potential mate if they pass all or nearly all of the agent's aspiration checks

return((rowSums(aspiration)^15))

}

if(agentalgorithm == 3){

#Enters each potential mate's traits into a cubic formula with the agent's ideal preferences as slopes.

return(apply(targettraits,1,function(x) sum(agentprefs[seq(1,92,4)]*x+agentprefs[seq(2,92,4)]*x^2+agentprefs[seq(3,92,4)]*x^3+agentprefs[seq(4,92,4)])))

}

if(agentalgorithm == 4){

#For each trait, the agent checks whether the potential mate's trait value is within the acceptable range. A potential mate gets an attractiveness point for each aspiration check they pass.

aspiration<-sapply(1:23,simplify=T,function(x) abs(as.numeric(agentprefs[((x*4)-1)])-targettraits[,x]) <= abs(as.numeric(agentprefs[(x*4)])))

#Determines the number of aspiration checks the potential mate passed

aspiration<-(rowSums(aspiration))

#Multiplies the agent's preferences by each mate's traits, adds an intercept, and sums across traits

regatt<-apply(targettraits,1,function(x) sum(x*agentprefs[seq(1,92,4)]+agentprefs[seq(2,92,4)]))

#An agent's attractiveness will be 0 unless it passes all aspiration checks. Otherwise, their attractiveness will be based on the regression algorithm

att<-rep(0,nrow(targettraits))

att[aspiration==23]<-regatt[aspiration==23]

return(att)

}

if(agentalgorithm == 5){

#Renames the agent preferences and traits so they can be rbound

agentprefs<-agentprefs[seq(1,92,4)]

names(agentprefs)<-1:23

colnames(targettraits)<-1:23

#Calculates the inverse euclidean distance between the agent's preferences and each mate's traits

#I add a small value to each distance just in case a distance is ever 0

return(apply(targettraits,1,function(x) 1/(dist(rbind(agentprefs,x))+1e-15)))

}

if(agentalgorithm == 6){

#Renames the agent preferences and traits so they can be rbound

eucprefs<-agentprefs[seq(1,92,4)]

names(eucprefs)<-1:23

colnames(targettraits)<-1:23

#Calculates the inverse euclidean distance between the agent's preferences and each mate's traits

#I add a small value to each distance just in case a distance is ever 0

eucatt<-apply(targettraits,1,function(x) 1/(dist(rbind(eucprefs,x))+1e-15))

#For each trait, the agent checks whether the potential mate's trait value is within the acceptable range. A potential mate gets an attractiveness point for each aspiration check they pass.

aspiration<-sapply(1:23,simplify=T,function(x) abs(as.numeric(agentprefs[((x*4)-2)])-targettraits[,x]) <= abs(as.numeric(agentprefs[((x*4)-1)])))

#Determines the number of aspiration checks the potential mate passed

aspiration<-(rowSums(aspiration))

#An agent's attractiveness will be 0 unless it passes all aspiration checks. Otherwise, their attractiveness will be based on the Euclidean algorithm

att<-rep(0,nrow(targettraits))

att[aspiration==23]<-eucatt[aspiration==23]

return(att)

}

if(agentalgorithm == 7){

#Generates a random attraction value for each potential mate

return(att<-runif(nrow(targettraits),0,1))

}

}

#Reproduction#

reproduceson<-function(mother,father){

#Mother is the traits, preferences, fitness points, algorithm, and sex of the mother

#Father is the traits, preferences, fitness points, algorithm, and sex of the father

#Sons inherit sexually dimorphic traits and preferences from their fathers. Sexually monomorphic traits and preferences have an equal chance of being inherited from either parent

traits<-c(ifelse(runif(5,0,1)>0,1,0),ifelse(runif(18,0,1)>.5,1,0))

traits<-ifelse(traits==1,as.numeric(father),as.numeric(mother))

preferences<-c(ifelse(runif(20,0,1)>0,1,0),ifelse(runif(73,0,1)>.5,1,0))

preferences<-ifelse(preferences==1,as.numeric(father[24:116]),as.numeric(mother[24:116]))

#Add a small amount of variability to traits and preferences

traits<-traits+rnorm(length(traits),0,.5)

preferences<-preferences+rnorm(length(preferences),0,.5)

#Create the son

son<-c(traits,preferences,as.numeric(father[117]))

#Sons after that have an equal chance of inheriting either parent's algorithm

son<-c(son,ifelse(runif(1,0,1)>.5,as.numeric(father[118]),as.numeric(mother[118])))

#Constrain traits appropriately

son[1:23]<-ifelse(son[1:23]>7,7,son[1:23])

son[1:23]<-ifelse(son[1:23]<1,1,son[1:23])

#Calculate the son's actual fitness points

son[116]<-fitnesspointscalc(son[1:23],malefitvec)

return(son)

}

reproducedaughter<-function(mother,father){

#Mother is the traits, preferences, fitness points, algorithm, and sex of the mother

#Father is the traits, preferences, fitness points, algorithm, and sex of the father

#Daughters inherit sexually dimorphic traits and preferences from their mothers. Sexually monomorphic traits and preferences have an equal chance of being inherited from either parent

traits<-c(ifelse(runif(5,0,1)>0,1,0),ifelse(runif(18,0,1)>.5,1,0))

traits<-ifelse(traits==1,as.numeric(mother),as.numeric(father))

preferences<-c(ifelse(runif(20,0,1)>0,1,0),ifelse(runif(73,0,1)>.5,1,0))

preferences<-ifelse(preferences==1,as.numeric(mother[24:116]),as.numeric(father[24:116]))

#Add a small amount of variability to traits and preferences

traits<-traits+rnorm(length(traits),0,.5)

preferences<-preferences+rnorm(length(preferences),0,.5)

#Create the daughter

daughter<-c(traits,preferences,as.numeric(mother[117]))

#Daughters after that have an equal chance of inheriting either parent's algorithm

daughter<-c(daughter,ifelse(runif(1,0,1)>.5,as.numeric(mother[118]),as.numeric(father[118])))

#Constrain traits appropriately

daughter[1:23]<-ifelse(daughter[1:23]>7,7,daughter[1:23])

daughter[1:23]<-ifelse(daughter[1:23]<1,1,daughter[1:23])

#Calculate the daughter's actual fitness points

daughter[116]<-fitnesspointscalc(daughter[1:23],femalefitvec)

return(daughter)

}

#Standardize an entire matrix, because scale() apparently will only standardize columns...

matrixstandardize<-function(matrix,newmean,newsd){

#Kludgey fix to avoid problems when an algorithm goes extinct

if(!is.na(sd(matrix))){

#Kludgey fix to avoid problems when all agents produce the same attraciton value (can happen with the aspiration algorithm)

if(sd(matrix)>0){

zmat<-(matrix-mean(matrix))/sd(matrix)

return(zmat*newsd+newmean)

} else{

return(matrix(newmean,nrow(matrix),ncol(matrix)))

}

} else{

return()

}

}

######Model Start######

#The below is for looping model runs, if desired

modelloops<-5

for(m in 1:modelloops){

######Agent Generation######

###Fitness Vector###

basefitvec<-matrix(1:7,7,18)

basefitvec<-apply(basefitvec,2,function(x) sample(x,size=length(x),replace=F))

malefitvec<-matrix(1:7,7,5)

malefitvec<-apply(malefitvec,2,function(x) sample(x,size=length(x),replace=F))

malefitvec<-cbind(malefitvec,basefitvec)

colnames(malefitvec)<-paste('trait',1:ncol(malefitvec),sep="")

femalefitvec<-matrix(1:7,7,5)

femalefitvec<-apply(femalefitvec,2,function(x) sample(x,size=length(x),replace=F))

femalefitvec<-cbind(femalefitvec,basefitvec)

colnames(femalefitvec)<-paste('trait',1:ncol(femalefitvec),sep="")

#Algorithms:

#1 = regression

#2 = aspiration

#3 = polynomial regression

#4 = threshold regression

#5 = euclidean

#6 = threshold euclidean

#7 = random

#Generate agents

regmales<-agentgenerate(50,1,1)

regfemales<-agentgenerate(50,0,1)

aspirationmales<-agentgenerate(50,1,2)

aspirationfemales<-agentgenerate(50,0,2)

polymales<-agentgenerate(50,1,3)

polyfemales<-agentgenerate(50,0,3)

threshregmales<-agentgenerate(50,1,4)

threshregfemales<-agentgenerate(50,0,4)

euclidmales<-agentgenerate(50,1,5)

euclidfemales<-agentgenerate(50,0,5)

thresheuclidmales<-agentgenerate(50,1,6)

thresheuclidfemales<-agentgenerate(50,0,6)

randommales<-agentgenerate(50,1,7)

randomfemales<-agentgenerate(50,0,7)

#Compile the parent dataframes, separated by sex

males<-rbind(regmales,aspirationmales,polymales,threshregmales,euclidmales,thresheuclidmales,randommales)

females<-rbind(regfemales,aspirationfemales,polyfemales,threshregfemales,euclidfemales,thresheuclidfemales,randomfemales)

#Give each agent a unique PIN

males$PIN<-sample(1:nrow(males),nrow(males),replace=F)

females$PIN<-sample(nrow(males):(nrow(males)+nrow(females)),nrow(females),replace=F)

########## Life Cycle ##########

#Life cycle begins here

#Iterations at this level represent generations, set below.

#Also can create dataframes here for storing data of interest

generations<-200

algorithmfreq<-data.frame(matrix(0,generations+1,14))

algorithmfreq[1,]<-c(nrow(males[males$algorithm==1,]),nrow(females[females$algorithm==1,]),nrow(males[males$algorithm==2,]),nrow(females[females$algorithm==2,]),nrow(males[males$algorithm==3,]),nrow(females[females$algorithm==3,]),nrow(males[males$algorithm==4,]),nrow(females[females$algorithm==4,]),nrow(males[males$algorithm==5,]),nrow(females[females$algorithm==5,]),nrow(males[males$algorithm==6,]),nrow(females[females$algorithm==6,]),nrow(males[males$algorithm==7,]),nrow(females[females$algorithm==7,]))

for(g in 1:generations){

######Computing Attraction######

#Males

#Calculate how attracted each male is to each female

maleattmatrix<-t(apply(males,1,function(q) attraction(q[24:115],q[118],females[,1:23])))

#Scale attraction

#Algorithms:

#1 = regression

#2 = aspiration

#3 = polynomial regression

#4 = threshold regression

#5 = euclidean

#6 = threshold euclidean

#7 = random

#The attraction values of all algorithms are scaled so that their mean equals 50 and their standard deviation = 15. Below threshold values are maintained as zeros.

maleattmatrix[males$algorithm==1,]<-matrixstandardize(maleattmatrix[males$algorithm==1,],50,15)

maleattmatrix[males$algorithm==2,]<-matrixstandardize(maleattmatrix[males$algorithm==2,],50,15)

maleattmatrix[males$algorithm==3,]<-matrixstandardize(maleattmatrix[males$algorithm==3,],50,15)

maleattmatrix[males$algorithm==4,]<-ifelse(maleattmatrix[males$algorithm==4,]>0,matrixstandardize(maleattmatrix[males$algorithm==4,],50,15),0)

maleattmatrix[males$algorithm==5,]<-matrixstandardize(maleattmatrix[males$algorithm==5,],50,15)

maleattmatrix[males$algorithm==6,]<-ifelse(maleattmatrix[males$algorithm==6,]>0,matrixstandardize(maleattmatrix[males$algorithm==6,],50,15),0)

maleattmatrix[males$algorithm==7,]<-matrixstandardize(maleattmatrix[males$algorithm==7,],50,15)

#Females

#Calculate how attracted each female is to each male

femaleattmatrix<-t(apply(females,1,function(q) attraction(q[24:115],q[118],males[,1:23])))

#Scale attraction

#Algorithms:

#1 = regression

#2 = aspiration

#3 = polynomial regression

#4 = threshold regression

#5 = euclidean

#6 = threshold euclidean

#7 = random

#The attraction values of all algorithms are scaled so that their mean equals 50 and their standard deviation = 15. Below threshold values are maintained as zeros.

femaleattmatrix[females$algorithm==1,]<-matrixstandardize(femaleattmatrix[females$algorithm==1,],50,15)

femaleattmatrix[females$algorithm==2,]<-matrixstandardize(femaleattmatrix[females$algorithm==2,],50,15)

femaleattmatrix[females$algorithm==3,]<-matrixstandardize(femaleattmatrix[females$algorithm==3,],50,15)

femaleattmatrix[females$algorithm==4,]<-ifelse(femaleattmatrix[females$algorithm==4,]>0,matrixstandardize(femaleattmatrix[females$algorithm==4,],50,15),0)

femaleattmatrix[females$algorithm==5,]<-matrixstandardize(femaleattmatrix[females$algorithm==5,],50,15)

femaleattmatrix[females$algorithm==6,]<-ifelse(femaleattmatrix[females$algorithm==6,]>0,matrixstandardize(femaleattmatrix[females$algorithm==6,],50,15),0)

femaleattmatrix[females$algorithm==7,]<-matrixstandardize(femaleattmatrix[females$algorithm==7,],50,15)

######Mate Selection######

#Mutual attraction matrix

#Computes the mutual attraction matrix--how mutually attracted each possible couple would be.

mutattmatrix<-as.data.frame(cbind(males$PIN,maleattmatrix*t(femaleattmatrix)))

#Renames the columns to be the female PIN values

colnames(mutattmatrix)<-c("MPIN",females$PIN)

#A blank dataframe for storing the paired couples

pairs<-matrix(,min(nrow(males),nrow(females)),(ncol(males)+ncol(females)))

pairs<-as.data.frame(pairs)

#Iterates through the mutual attraction matrix. Will only iterate once for each member of the scarcer sex (that way all possible pairs are formed)

for(i in 1:min(nrow(males),nrow(females))){

#Determines the location of the maximum mutual attraction value in the matrix

maxmut<-which(mutattmatrix[,2:ncol(mutattmatrix)]==max(mutattmatrix[,2:ncol(mutattmatrix)]),arr.ind=T)

#Kludgey fix. When only one female remains, R says there are no columns (because there's only one column of females). In that case, we assume it meant the column is 1 (the only remaining female)

if(ncol(mutattmatrix)==2){maxmut<-cbind(as.vector(maxmut),1)}

#If there is more than one location equal to the maximum, picks a random location

if(length(maxmut)/2 > 1){maxmut<-maxmut[sample(nrow(maxmut),1,replace=F),]}

#Determines which male and which female were actually paired

pairedmale<-males[males$PIN==mutattmatrix[maxmut[1],1],]

pairedfemale<-females[females$PIN==colnames(mutattmatrix)[(maxmut[2]+1)],]

#Places the paired male and female in the pairs dataframe

pairs[i,]<-cbind(pairedfemale,pairedmale)

#Removes the paired male and female from the mutual attraction matrix

mutattmatrix<-mutattmatrix[-maxmut[1],-(maxmut[2]+1)]

#Subtracts .25 fitness points from all agents who remain unpaired. If statement is another kludgey fix that prevents problems when the mutual attraction matrix is empty or unidimensional

if(identical(mutattmatrix,numeric(0))==F & is.null(ncol(mutattmatrix))==F){

males$fitpoints[males$PIN %in% mutattmatrix[,1]]<-males$fitpoints[males$PIN %in% mutattmatrix[,1]]-.25

females$fitpoints[females$PIN %in% colnames(mutattmatrix)]<-females$fitpoints[females$PIN %in% colnames(mutattmatrix)]-.25

}

}

#Just names the pairs matrix appropriately so it's easier to read. Probably unnecessary.

colnames(pairs)<-c(colnames(females),colnames(males))

######Reproduce######

#Empty matrix for storing offspring

maleoffspring<-matrix(,,118)

femaleoffspring<-matrix(,,118)

#If the parents wind up with negative fitness points, they get reset to 0

pairs[,116]<-sapply(pairs[,116],simplify=T,function(x) ifelse(x<0,0,x))

pairs[,235]<-sapply(pairs[,235],simplify=T,function(x) ifelse(x<0,0,x))

#Iterate through each couple

for(i in 1:nrow(pairs)){

#Determine how many offspring each couple should have based on their fitness points. One offspring costs 40 fitness points.

offspringnumber<-round((pairs[i,116]+pairs[i,235])/40,0)

#Randomly determine the sex of their offspring

offspringsexes<-rbinom(offspringnumber,1,.5)

#Split into two vectors for easy looping of the reproduction functions

maleoffspringnum<-offspringsexes[offspringsexes==1]

femaleoffspringnum<-offspringsexes[offspringsexes==0]

#Just separate these out now to prevent clutter later.

mother<-pairs[i,1:119]

father<-pairs[i,120:238]

#For each male offspring, generate a male offspring and place it in the maleoffspring matrix

if(length(maleoffspringnum)>0){

maleoffspring<-rbind(maleoffspring,t(sapply(maleoffspringnum,function(x) reproduceson(mother,father))))

}

#For each female offspring, generate a female offspring and place it in the femaleoffspring matrix

if(length(femaleoffspringnum)>0){

femaleoffspring<-rbind(femaleoffspring,t(sapply(femaleoffspringnum,function(x) reproducedaughter(mother,father))))

}

}

#Drop the initial row of NAs.

maleoffspring<-maleoffspring[-1,]

femaleoffspring<-femaleoffspring[-1,]

#Create one dataframe of offspring (and actually make it a dataframe), which will make imposing the population cap easier in a moment.

offspring<-as.data.frame(rbind(femaleoffspring,maleoffspring))

#Name everything for ease of use. Probably not necessary.

colnames(offspring)<-colnames(females)[1:118]

######Death######

#If the offspring population is above the cap, kill a random sample of offspring to lower the population to cap

if(nrow(offspring)>700){

offspring<-offspring[sample(nrow(offspring),700,replace=F),]

}

#Give each offspring a PIN

offspring$PIN<-sample(1:nrow(offspring),nrow(offspring),replace=F)

#Save the offspring over the parent dataframes. This kills the parents.

males<-subset(offspring,offspring$sex==1)

females<-subset(offspring,offspring$sex==0)

######Data Saving######

#Here is where I save whatever data is of interest. Population sizes for each algorithm, in this case.

algorithmfreq[g+1,]<-c(nrow(males[males$algorithm==1,]),nrow(females[females$algorithm==1,]),nrow(males[males$algorithm==2,]),nrow(females[females$algorithm==2,]),nrow(males[males$algorithm==3,]),nrow(females[females$algorithm==3,]),nrow(males[males$algorithm==4,]),nrow(females[females$algorithm==4,]),nrow(males[males$algorithm==5,]),nrow(females[females$algorithm==5,]),nrow(males[males$algorithm==6,]),nrow(females[females$algorithm==6,]),nrow(males[males$algorithm==7,]),nrow(females[females$algorithm==7,]))

}

#Algorithms:

#1 = regression

#2 = aspiration

#3 = polynomial regression

#4 = threshold regression

#5 = euclidean

#6 = threshold euclidean

#7 = random

colnames(algorithmfreq)<-c("malesreg","femalesreg","malesapiration","femalesaspiration","malespoly","femalespoly","malesthreshreg","femalesthresreg","maleseuclid","femaleseuclid","malesthrescheuclid","femalesthresheuclid","malesrandom","femalesrandom")

#Compile the algorithm frequencies into one dataframe

freqdata<-data.frame("algorithm"=c("Regression","Aspiration","Polynomial Regression","Threshold Regression","Euclidean","Threshold Euclidean","Random"))

freqdata<-cbind(freqdata,apply(algorithmfreq,1,function(x) c(sum(x[1],x[2]),sum(x[3],x[4]),sum(x[5],x[6]),sum(x[7],x[8]),sum(x[9],x[10]),sum(x[11],x[12]),sum(x[13],x[14]))))

colnames(freqdata)<-c("algorithm",paste("generation",1:(generations+1),sep=""))

#Create a unique filename so I don't overwrite old results.

#Name format is "Algorithm Frequencies MonthDayYear HourMinute"

#path<-"/Algorithm Frequencies "

path<-"/Algorithm Frequencies "

format<-".csv"

date<-format(Sys.time(),format="%m%d%Y %H%M")

file<-file.path(paste0(path,date,format))

write.csv(freqdata,file=file)

}

#Plot data overall

library(reshape)

library(ggplot2)

#Load in the data (be sure the working directory is set to where the model run files are saved)

generations<-200

#Compile the individual model run files into one big dataframe

data<-lapply(list.files(),read.csv)

data<-lapply(data,function(x) x[,2:(generations+3)])

simdata<-NULL

simdata<-do.call(rbind,data)

#COmpute the average population size of each algorithm in each generation as well as the 95% confidence intervals.

graphdata<-data.frame("algorithm"=c("Simple Regression","Aspiration","Polynomial Regression","Threshold Regression","Euclidean","Threshold Euclidean","Random"))

graphdata<-cbind(graphdata,rbind(colMeans(simdata[simdata$algorithm=="Regression",2:ncol(simdata)]),colMeans(simdata[simdata$algorithm=="Aspiration",2:ncol(simdata)]),colMeans(simdata[simdata$algorithm=="Polynomial Regression",2:ncol(simdata)]),colMeans(simdata[simdata$algorithm=="Threshold Regression",2:ncol(simdata)]),colMeans(simdata[simdata$algorithm=="Euclidean",2:ncol(simdata)]),colMeans(simdata[simdata$algorithm=="Threshold Euclidean",2:ncol(simdata)]),colMeans(simdata[simdata$algorithm=="Random",2:ncol(simdata)])))

generationse<-as.matrix(cbind(graphdata$algorithm,rbind(apply(simdata[simdata$algorithm=="Regression",-1],2,function(x) qt(.975,(nrow(simdata)/7)-2)*(sd(x)/sqrt(length(x)))),apply(simdata[simdata$algorithm=="Aspiration",-1],2,function(x) qt(.975,(nrow(simdata)/7)-2)*(sd(x)/sqrt(length(x)))),apply(simdata[simdata$algorithm=="Polynomial Regression",-1],2,function(x) qt(.975,(nrow(simdata)/7)-2)*(sd(x)/sqrt(length(x)))),apply(simdata[simdata$algorithm=="Threshold Regression",-1],2,function(x) qt(.975,(nrow(simdata)/7)-2)*(sd(x)/sqrt(length(x)))),apply(simdata[simdata$algorithm=="Euclidean",-1],2,function(x) qt(.975,(nrow(simdata)/7)-2)*(sd(x)/sqrt(length(x)))),apply(simdata[simdata$algorithm=="Threshold Euclidean",-1],2,function(x) qt(.975,(nrow(simdata)/7)-2)*(sd(x)/sqrt(length(x)))),apply(simdata[simdata$algorithm=="Random",-1],2,function(x) qt(.975,(nrow(simdata)/7)-2)*(sd(x)/sqrt(length(x)))))))

generationleb<-cbind(graphdata$algorithm,graphdata[,-1]-generationse[,-1])

generationheb<-cbind(graphdata$algorithm,graphdata[,-1]+generationse[,-1])

#Prevent error bars lower than 0. This always prints a warning because of applying a logical operator to a string (the algorithm names), so I suppress that warning.

suppressWarnings(generationleb[generationleb<0]<-0)

#Re-organize the data into longform for plotting

graphdata<-melt(graphdata,id="algorithm")

generationleb<-melt(generationleb,id="graphdata$algorithm")

generationheb<-melt(generationheb,id="graphdata$algorithm")

colnames(graphdata)<-c("algorithm","generation","mean")

graphdata$leb<-generationleb$value

graphdata$heb<-generationheb$value

graphdata<-graphdata[order(graphdata$algorithm),]

graphdata$generation<-rep(1:(generations+1),7)

#Plot

q<-qplot(generation,mean,color=algorithm,data=graphdata,geom="line",xlab="Generation",ylab="Population")+scale_color_discrete(name="Algorithm")+theme_classic()

limits<-aes(ymax=graphdata$heb,ymin=graphdata$leb)

q+geom_errorbar(limits)

#Blank matrices for storing data on algorithm performances

dominantalgorithm<-matrix(0,7,1)

algorithmfix<-matrix(0,7,1)

algorithmextinct<-matrix(0,7,1)

#Loop through the model runs

for(i in 1:length(data)){

#Pull out just the population data from the final generation for the current run

checkdata<-simdata[(i*7-6):(i*7),202]

#Determine which algorithm had the highest population in the final generation

maxrun<-which(checkdata==max(checkdata))

#Add one to that algorithm's tally of maximum population size

dominantalgorithm[maxrun]<-dominantalgorithm[maxrun]+1

#Determine whether the dominant algorithm also fixed in that model run. Give it a point if it did.

algorithmfix[maxrun]<-ifelse(sum(checkdata[-maxrun])==0,algorithmfix[maxrun]+1,algorithmfix[maxrun])

#Determine which algorithms went extinct, and give them a point for each model in which they went extinct

extinctrun<-which(checkdata==0)

algorithmextinct[extinctrun]<-algorithmextinct[extinctrun]+1

}

#Compile all of the data, as well as calculate the percentage of runs in which each algorithm was dominant, fixed, or went extinct

algperf<-data.frame("algorithm"=c("Regression","Aspiration","Polynomial Regression","Threshold Regression","Euclidean","Threshold Euclidean","Random"))

algperf$dominant<-as.vector(dominantalgorithm)

algperf$dominantpercent<-as.vector(dominantalgorithm/length(data))

algperf$fixed<-as.vector(algorithmfix)

algperf$fixedpercent<-as.vector(algorithmfix/length(data))

algperf$extinct<-as.vector(algorithmextinct)

algperf$extinctpercent<-as.vector(algorithmextinct/length(data))
